# Neural Substrates of Visual Perception and Working Memory: Two Sides of the Same Coin or Two Different Coins?

**DOI:** 10.3389/fncir.2021.764177

**Published:** 2021-11-26

**Authors:** Megan Roussy, Diego Mendoza-Halliday, Julio C. Martinez-Trujillo

**Affiliations:** ^1^Department of Physiology and Pharmacology, Schulich School of Medicine & Dentistry, Robarts Research Institute, University of Western Ontario, London, ON, Canada; ^2^McGovern Institute for Brain Research, Massachusetts Institute of Technology, Cambridge, MA, United States

**Keywords:** visual perception, working memory, persistent activity, prefrontal cortex, visual system

## Abstract

Visual perception occurs when a set of physical signals emanating from the environment enter the visual system and the brain interprets such signals as a percept. Visual working memory occurs when the brain produces and maintains a mental representation of a percept while the physical signals corresponding to that percept are not available. Early studies in humans and non-human primates demonstrated that lesions of the prefrontal cortex impair performance during visual working memory tasks but not during perceptual tasks. These studies attributed a fundamental role in working memory and a lesser role in visual perception to the prefrontal cortex. Indeed, single cell recording studies have found that neurons in the lateral prefrontal cortex of macaques encode working memory representations via persistent firing, validating the results of lesion studies. However, other studies have reported that neurons in some areas of the parietal and temporal lobe—classically associated with visual perception—similarly encode working memory representations via persistent firing. This prompted a line of enquiry about the role of the prefrontal and other associative cortices in working memory and perception. Here, we review evidence from single neuron studies in macaque monkeys examining working memory representations across different areas of the visual hierarchy and link them to studies examining the role of the same areas in visual perception. We conclude that neurons in early visual areas of both ventral (V1-V2-V4) and dorsal (V1-V3-MT) visual pathways of macaques mainly encode perceptual signals. On the other hand, areas downstream from V4 and MT contain subpopulations of neurons that encode both perceptual and/or working memory signals. Differences in cortical architecture (neuronal types, layer composition, and synaptic density and distribution) may be linked to the differential encoding of perceptual and working memory signals between early visual areas and higher association areas.

## 1. Introduction

### 1.1. Are Working Memory and Perception Two Distinct Cognitive Functions?

Visual perception is defined as the ability to interpret the surrounding environment from electromagnetic signals entering the retinas. Visual perception occurs when neurons across different areas of the visual system are activated by retinal inputs and the brain produces “a percept” or interpretation of the physical reality (e.g., seeing a red shirt) (Chalupa and Werner, 2003). Visual working memory is the ability to remember and manipulate, for short periods of time, an interpretation of the physical reality when the corresponding physical signals are no longer entering the retinas (Baddeley, 2010) (e.g., the mental image or memory of the same red shirt). Perhaps the best operational distinction between visual perception and working memory is that the former is linked to the flow of visual inputs, while the latter is not. The distinction between perceptual and mnemonic states seems intuitive. Indeed, a typical human subject can distinguish when they “see” an image of a red shirt (perceptual) and when they “remember” an image of a red shirt (mnemonic). Thus, for typical individuals, the mental states corresponding to visual perception and working memory are different and distinguishable.

It is important to clarify that perception is not always a lawful reflection of the physical properties of stimuli. Phenomena such as perceptual illusions have taught us that perception is a creative process, and under particular circumstances of ambiguity, we could “misinterpret” the physical environment or even interpret the same environment in multiple ways (Todorović, 2020). However, we would argue that in general, perception reflects the physical reality in a predictable manner. Therefore, in the current review, we refer to perception as a predictable and stable process and exclude cases of perceptual illusions or variations (Foster, 2011). We focus on the distinction between the physical presence of an object (visual perception) and the mental image of the same object when unavailable to the senses (mnemonic representation).

A somewhat related review of this topic largely based on findings from human experiments using non- invasive signal measurement techniques has been recently published (Dijkstra et al., 2021). In this current review, we primarily refer to data collected in experiments using invasive techniques in non-human primates such as lesion studies and electrophysiological recordings. We make the reasonable axiomatic assumption that anthropoid non-human primates with a developed visual system and brain areas that have human homologs (Petrides, 2005) use perception and working memory as part of their cognitive repertoire (Beran et al., 2016).

The distinction between working memory and perceptual functions can be traced to lesions studies conducted more than a century ago in humans and animals. They reported that damage to certain brain areas can produce selective deficits of working memory while sparing visual perception (reviewed in the next section). However, more recent studies have reported co-existence of signal correlates of visual perception and working memory across brain areas and have questioned the segregation of the neural substrates for these two functions in the brain (reviewed in the section “Dissociating Visual Working Memory and Perception: Electrophysiological Studies of Single Neurons Across Brain Areas”). Influential in this latter view, have been findings of functional imaging and EEG/MEG studies in human subjects (Dijkstra et al., 2021).

On a cautionary note, we have found that the diversity of techniques used to record brain signals in humans and non-human primates and that of paradigms (tasks) used to explore working memory and perception makes it difficult to examine the relationship between the neural correlates of the two functions across species. This is in part because different techniques used in humans and non-human primates explore different spatial and temporal scales of brain activity and record different types of signals. It is therefore difficult to reconcile the results of studies in different species. In this review, we have taken a focused approach to examine reports mainly from studies in non-human primates using different methodologies to study working memory coding along areas of the visual processing pathways and its relationship to visual perception. We also assume that over the short temporal scales of perception and working memory, action potentials are the central elements of information coding and transmission between neurons and neuronal networks over distances that extend beyond synapses. Therefore, we concentrate on studies that have directly recorded action potentials from neurons or neuronal populations during behavioral tasks that involve visual perception and working memory.

## 2. Main

### 2.1. Dissociating Visual Working Memory and Perception: Lesion Studies

The idea that perceptual and mnemonic representations are separable in the brain originated by investigations into patients with localized cortical damage. Although they did not directly measure working memory, early case studies describe independent impairments in top-down driven representations (visual imagery) or perception. Charcot and Bernard first described a patient in 1883 that could identify objects but was neither able to form mental representations of these objects nor envision them from memory (Charcot and Bernard, 1883). The opposite deficit has also been described in which patients are unable to perceive objects yet can describe them in detail based on clear mental representations. A well-known case of this, described in patient C.K, was presented by Behrmann and colleagues in the early 1990's. C.K was unable to identify either simple or complex items but was able to produce clear and detailed drawings of those same items (Behrmann et al., 1994).

Early lesion studies in non-human primates supported the dissociation between working memory and perception. Jacobsen (1936) conducted a series of lesion experiments in the prefrontal cortex (PFC) of different species of non-human primates [*Macaca mulatta* (rhesus macaque), *Cercocebus torquatus* (mangabey), and *Papio papio* (baboon)] and noticed that the lesions produced selective performance deficits in delayed response tasks, where animals had to remember the locations or features of objects for a short period of time. Importantly, the animals could perform other perceptual tasks without major difficulty (Jacobsen, 1936). These results suggested that lesions of the PFC affect mainly working memory while sparing perception. In another study, Chow, Blum and Blum conducted lesion experiments of the posterior association areas of the parieto-occipital temporal region and the prefrontal areas close to the frontal pole in macaque monkeys (Chow et al., 1951). They found that posterior lesions did not substantially affect performance in a delayed response task. On the other hand, prefrontal lesions did affect the animals' performance without substantially affecting other discrimination abilities. They concluded that the PFC plays a selective role in the delayed aspects of the task.

In 1952, Harlow and colleagues reported two distinct deficits associated with lesions of the posterior cortices and anterior (prefrontal) cortices in macaque monkeys. The animals with posterior lesions had stronger deficits in discrimination tasks, whereas animals with anterior prefrontal lesions had stronger deficits in delayed response tasks (Harlow et al., 1952). Curiously, lesions to the posterior parietal cortex have little effect on the performance of delayed response tasks. In the case of complete and bilateral posterior parietal cortex lesions, visuospatial information may possibly arrive to PFC through alternate connections (i.e., anterior/posterior cingulate cortex) or through connections to preoccipital regions (i.e., dorsomedial area DP), via the occipitofrontal fascicle (Selemon and Goldman-Rakic, 1988; Yeterian and Pandya, 2010; Yeterian et al., 2012; Arnsten, 2013).

In 1952, Pribram and coworkers described that lesion of the PFC in baboons (*Papio papio*) also produced performance deficits in delayed response tasks. Dorsolateral lesioned animals had greater alterations in all tasks compared to ventromedial lesioned animals (Pribram et al., 1952). In 1969, a study by Butters and Pandya (1969) reported a more specific finding concerning the role of the PFC in working memory tasks. They compared the performance of lesioned and control rhesus macaques in delayed alternation tasks. Lesions included bilateral inferior parietal cortex lesions and three types of prefrontal lesions around the principal sulcus. Animals with lesions of the anterior and posterior thirds of the principal sulcus as well as periarcuate and parietal lesions could re-learn the delay alternation task but animals with lesions of the central part of the arcuate sulcus could not re-learn the task and showed permanent deficits. A later study by Warren and Divac (1972) demonstrated that the effect of principal sulcus lesions extends to delayed response tasks.

Importantly, decades earlier, Malmo (1942) and Orbach and Fischer (1959) reported the importance of the PFC in maintaining working memory representations in the presence of irrelevant incoming visual signals. Without PFC, stored mental representations can be disrupted by incoming sensory signals. These studies highlighted the importance of PFC to guard mental representations from distracters.

In 1960, Miles and Blomquist (1960) reported that lesions of the PFC in squirrel monkeys (*Samiri sciureus*), a new world primate, produced a similar syndrome as the one observed in the old world species. The syndrome consisted of hyperactivity, deficits in delayed response tasks, and no adverse effects on the ability to solve discrimination tasks when the stimulus was present. This study extends the observed effects of prefrontal lesions to new world monkeys, with a relatively less expanded PFC than their old world relatives (Passingham and Wise, 2012).

**Figure 1:**
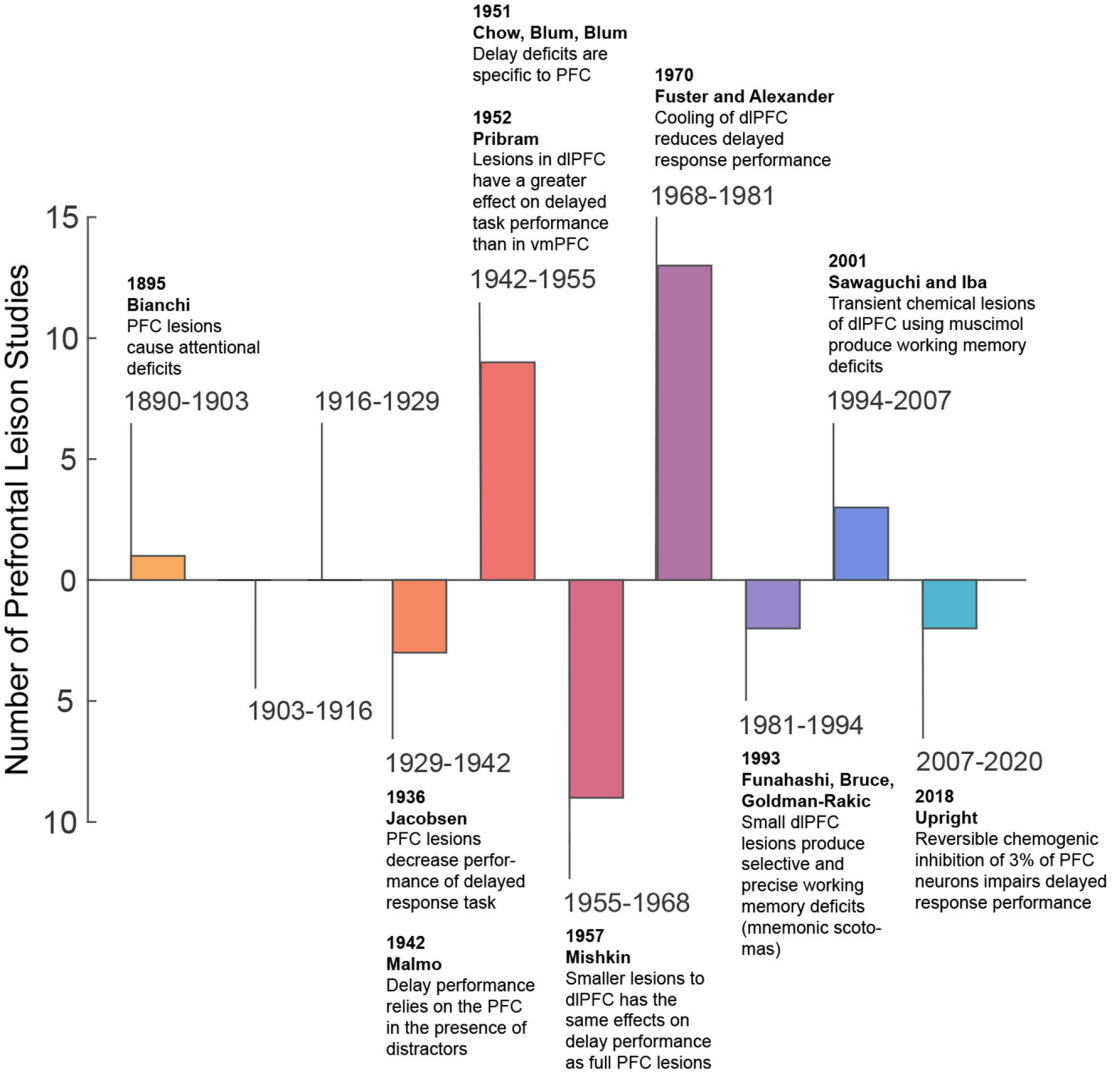
Summary of lesion studies.

More recently, in the second half of the twentieth century, spatially refined lesion and pharmaceutical inactivation studies in the PFC of macaque monkeys further demonstrated perturbation of visuospatial working memory representations and sparing of perceptual representations (Sawaguchi and Goldman-Rakic, 1991; Funahashi et al., 1993; Iba and Sawaguchi, 2003). This work introduced the concept of mnemonic scotoma, a deficit in remembering a certain spatial location during a delayed response task induced by inactivating small regions in the lateral prefrontal cortex (LPFC) (Funahashi et al., 1993). However, animals with mnemonic scotomas are able to make saccades to the region of the mnemonic scotoma when the target object is visually available. The latter not only confirmed the results of previous studies, but also emphasized a major role of the PFC in visual working memory and a lesser role in visual perception. Thus, from lesions studies, one may conclude the PFC is needed for maintaining information in working memory, but it is not essential for visual perception (i.e., when visual information remains available). Table 1 shows a summary of studies that explore the effects of lesions in perceptual and working memory tasks in non-human primates. Figure 1 provides a graphical summary of this information.

**Table 1:** Lesion studies.

**References**	**Species**	**Main finding**
Bianchi (1895)	*Papio cynocephalus*	Lesions of the frontal cortex resulted in attentional but not perceptual deficits. Concludes that the frontal lobes serve to fuse incoming sensory signals and motor output forming associative representations.
Jacobsen et al. (1935)	*Pan troglodytes*	Bilateral lesions of the prefrontal cortex diminished performance on a delayed response task.
Jacobsen (1936)	*Macaca mulattaCerocebus torquatusPapio papio*	Bilateral lesions of the prefrontal cortex diminished performance on a delayed response task.
Jacobsen and Nissen (1937)	*Macaca mulatta*	Bilateral lesions of the prefrontal cortex diminished performance on a delayed alternation task.
Malmo (1942)	*Macaca mulattaCerocebus torquatus*	Bilateral prefrontal lesions made animals more susceptible to extraneous stimuli occurring during the delay interval of a delayed response task.
Finan (1942)	*Cerocebus torquatus*	Bilateral prefrontal lesions decrease performance of a delayed response task. Pre-rewarded food increased performance.
Spaet and Harlow (1943)	*Macaca mulatta*	Bilateral prefrontal lesions created greater deficits in delayed reaction problems (non-spatial delayed reaction, spatial delayed reaction) than in stimulus-object discrimination problems.
Campbell and Harlow (1945)	*Macaca mulatta*	Bilateral lesions of the frontal cortex related in reduced performance on a spatial delayed response task. Performance differed based on recovery time from surgery.
Pribram (1950)	*Papio porcarius*	Bilateral lesion of the prefrontal cortex anterior to FEF decreased performance on a delayed response task. Insulin administration, cooling and fasting increased performance likely through increased reward value of the stimulus (food).
Chow et al. (1951)	*Macaca mulatta*	Animals with bilateral lesions of the prefrontal cortex showed similar performance deficits on a delayed reaction test as animals with prefrontal lesions and additional damage to parietal and temporal regions. Sedative drugs did not improve performance.
Harlow et al. (1952)	*Macaca mulatta*	Anterior and posterior lesions produce predominantly delay response and discrimination deficits respectively.
Pribram et al. (1952)	*Papio papio*	Dorsolateral lesions reduced performance on delayed response-type problems but showed little effect on visual-discrimination task performance. Two of the four animals with ventromedial lesions showed no change in task performance.
Blum (1952)	*Macaca mulatta*	Lesions to the ventrolateral and dorsal region produced smaller deficits in a visual and auditory delay reaction tasks while lesions in the midlateral region (region anterior to the arcuate sulcus) produced large deficits.
Mishkin and Pribram (1955)	Macaque (unknown)	Lesions to the anterolateral frontal cortex resulted in poor performance on a series of delayed alternation problems.
Mishkin and Pribram (1956)	*Macaca mulatta*	Animals with bilateral anterolateral prefrontal lesions were tested on a series of delayed response tasks. Lesions resulted in deficits in the performance of traditional delayed response tasks, but performance increased when traditional cues are replaced by non-positional cues.
Orbach (1956)	*Macaca mulatta*	Bilateral prefrontal lesions resulted in deficits in a delayed response task within hours after surgery. This deficit was present 14 days after surgery though there was a slight recovery in performance.
Rosvold and Delgado (1956)	*Macaca mulatta*	Stimulation in the region of the head of the caudate nucleus impaired alternation without affecting visual discrimination, as did tissue destruction in the same site.
Mishkin (1957)	*Macaca mulatta*	Lesions of the midlateral region of the prefrontal cortex (anterior to arcuate sulcus) produced a deficit in a delayed alternation task that was as severe as total anterior frontal lesions.
Orbach and Fischer (1959)	*Macaca mulatta*	Bilateral lesions of the frontal granular cortex reduced performance on a delayed response task. Performance in animals with lesions was further reduced with added light interruption. Retraining on the task after surgery did improve performance.
Miles and Blomquist (1960)	*Saimiri sciureus*	Bilateral frontal lesions result in reduced delayed response performance but show no change in discrimination learning.
Gross and Weiskrantz (1962)	*Macaca mulatta*	Lesions surrounding the principal sulcus resulted in greater impairment on delayed response tasks whereas frontal lesions excluding tissue surrounding the principal sulcus resulted in greater impairment on auditory-discrimination tasks. Lesions in either area did not affect performance of a visual-discrimination task.
Tucker and Kling (1967)	*Macaca mulatta Macaca speciosa*	Bilateral lesions of the dorsolateral frontal granular cortex at either the 35th postnatal day or 3 years of age showed similar deficits in a delayed alternation task but performance on a delayed response task was better in animals with earlier lesions.
Butters and Pandya (1969)	*Macaca mulatta*	Bilateral lesions were performed in the anterior, middle, or posterior thirds of the principal sulcus, of the periarcuate prefrontal region, or of the inferior parietal lobule. Lesions within the middle third of the principal sulcus produced deficits on a delayed alternation task whereas lesions in other regions had little effect.
Fuster and Alexander (1970)	*Macaca mulatta*	Performance of a delayed response task was impaired by bilateral cooling of the dorsolateral prefrontal cortex.
Goldman and Rosvold (1970)	*Macaca mulatta*	Lesions around the principal sulcus impaired performance on the spatial task with delay and lesions around the arcuate impaired performance on the spatial task without delay.
Goldman et al. (1971)	*Macaca mulatta*	Lesions to the dorsolateral prefrontal cortex and to regions along the principal sulcus resulted in deficits in both a spatial discrimination task and spatial delayed response task.
Stamm and Weber-Levine (1971)	*Macaca mulatta*	Total bilateral lesions of the dorsolateral prefrontal cortex and lesions of the banks and floor of the principal sulcus produced the greatest deficits on a delayed alternation task while lesions to the surrounding dorsolateral cortical strips produced smaller deficits.
Butters et al. (1971)	*Macaca mulatta*	Lesions were made in the superior and/or inferior banks of the middle third of principal sulcus. Lesions which involved both banks led to greater deficits in a spatial delayed alternation and place reversal task than lesions to either bank alone.
Warren and Divac (1972)	*Macaca mulatta*	Lesions of the middle third of principal sulcus decrease performance of a delayed response and delayed alternation task.
Fuster and Bauer (1974)	*Macaca mulatta*	Cooling of the prefrontal cortex reduced performance of a delayed matching-to-sample task with bilateral cooling having a greater effect than unilateral cooling. Cooling of the parietal cortex did not produce a deficit.
Oscar-Berman et al. (1975)	*Macaca mulatta*	Lesions to the dorsolateral prefrontal cortex produced greater deficits in a delayed response task than lesions to the ventrolateral orbito-frontal cortex but had a smaller impact on visual and auditory discrimination tasks.
Passingham (1975)	*Macaca mulatta*	Dorsal prefrontal lesions decreased performance of a spatial delayed alternation task but had little impact on a delayed matching task for colors. Ventral prefrontal lesions impaired performance on the delayed matching task for colors.
Bauer and Fuster (1976)	*Macaca mulatta*	Delayed matching and delayed response deficit from cooling dorsolateral prefrontal cortex in monkeys.
Mishkin and Manning (1978)	*Macaca mulatta*	Lesions surrounding the principal sulcus resulted in deficits on delayed spatial memory tasks but had little effect on three non-spatial tasks such as delayed object matching, and delayed color matching.
Brozoski et al. (1979)	*Macaca mulatta*	Depletion of prefrontal dopamine leads to deficits on delayed alternation but not visual pattern discrimination.
Sawaguchi and Goldman-Rakic (1991)	*Macaca mulatta*	Local injections of selective D1 receptor antagonists into the prefrontal cortex reduced performance of an oculomotor delayed response task but had no effect on performance of a visually guided saccade task.
Funahashi et al. (1993)	*Macaca mulatta*	Unilateral lesions of the dorsolateral prefrontal cortex produced the greatest deficits in an oculomotor delayed response task for contralateral targets. Deficits were not seen for a visually guided saccade task suggesting the existence of mnemonic scotomas.
Petrides (1995)	*Macaca nemestrina*	Lesions of the mid-dorsal part of the lateral produced deficits in non-spatial self-ordered and externally ordered working memory tasks. The number of remembered items influenced performance. Deficits were not seen after lesions of the posterior dorsolateral frontal cortex (surrounds the arcuate sulcus).
Petrides (2000)	*Macaca nemestrina*	Increasing the number of stimuli to be remembered during a visual working memory task impaired performance after mid-dorsolateral lesions but not after anterior inferotemporal lesions whereas the opposite was true after extending the duration of the delay period. Full lesion of the mid-dorsolateral region created greater deficits than lesions on area 9 alone.
Sawaguchi and Iba (2001)	*Macaca mulatta*	Local injection of muscimol into the dorsolateral prefrontal cortex produced deficits in an oculomotor delayed response task to specific and typically contralateral target locations. No deficits we identified for a visually guided saccade task.
Croxson et al. (2011)	*Macaca mulatta*	Selective lesions of cholinergic input to prefrontal cortex severely impaired on a spatial working memory task while leaving unimpaired decision-making and episodic memory.
Upright et al. (2018)	*Macaca mulatta*	Reversible chemogenetic inhibition of only 3% of prefrontal neurons is sufficient for impairing performance on a spatial delayed response task.

### 2.2. Dissociating Visual Working Memory and Perception Along the Visual Pathways

Departing from the accumulated evidence in early lesion studies in non-human primates (reviewed above) and the development of single cell recording techniques in behaving animals (Hubel, 1957), Fuster and Alexander (1971) recorded the responses of neurons in the LPFC and mediodorsal nucleus of the thalamus in macaque monkeys during delayed response tasks. They discovered cells in the LPFC that represent remembered locations and features of visual stimuli via persistent firing: an increase in firing rate above baseline tuned for the location of the items held in working memory. One important feature of persistent firing is that it occurs in the absence of sensory inputs, when the cue or sample stimulus disappears from the visual field—the so-called delay period of working memory tasks. An amount of controversy has been accumulating around the concept of persistent firing. For example, whether it is sustained during the entire delay period by single neurons or populations, or it has a temporal structure (e.g., oscillations in certain frequency bands) (Sreenivasan et al., 2014; Lundqvist et al., 2016, 2018; Constantinidis et al., 2018). In the original report Fuster and Alexander (1971) do not make considerations about the temporal structure of persistent firing in individual trials but used trial averages. Although clarifying the temporal structure of persistent firing is important to reveal the mechanisms of working memory coding, this review will not expand on this topic. We will consider persistent firing as increases in firing rate that encode the contents of working memory. The temporal structure of such changes may be variable in individual neurons and across tasks.

It must be noted that rodent models are commonly used to study short-term memory and delay activity has been reported in areas associated with rodent cognition, in particular the medial prefrontal cortex (Park et al., 2019; Ozdemir et al., 2020). Although experiments using rodent models have enriched our understanding of short-term memory mechanisms, the rodent visual system diverges from that of primates: rodents lack a granular prefrontal cortex making the comparison with primate brain regions problematic (Uylings et al., 2003; Passingham and Wise, 2012). Interareal connectivity between rat medial prefrontal cortex also diverges from primate lPFC in which it was shown to be more similar to primate premotor regions (Schaeffer et al., 2020), further complicating direct comparisons. The topic of similarities and differences between short term or working memory mechanisms in rodents (mice and rats) and primates necessitates an extensive discussion. Our review will therefore focus on experiments in primates.

We must also indicate here that we are not distinguishing different aspects of working memory in this review. What some believe makes working memory distinct is that it implies manipulation of information and not simply maintenance in its original form (Baddeley, 2010) (e.g., a mental rotation of an object or a reference frame transformation from retina-centered to space centered). However, physiological studies in non-human primates have not classically made that distinction, and refer to working memory in its maintenance aspect (Goldman-Rakic, 1995). We will continue this tradition here and acknowledge that work needs to be done to clarify this issue.

The initial results of Fuster and Alexander in the LPFC were confirmed by other studies (Kubota and Niki, 1971), thus supporting the hypothesis that the neural substrates of working memory is allocated to the LPFC in primates (areas 46/9, around the principal sulcus). Importantly, the existence of persistent firing pointed toward a different mechanism for working memory coding compared to the mechanisms of permanent synaptic storage for long-term memory (Eccles, 1986). The fundamental idea is that the memory is maintained as long as persistent firing is maintained; therefore, it dissipates when neurons stop firing. This matches the behavioral observations of working memory as a mechanism susceptible to temporal decay (Baddeley, 2010). It also agrees with the fact that most representations held in working memory are not transferred into long-term memory. Such a continuous transfer would be wasteful in many situations since many items held in working memory are “temporally useful” and therefore not needed to be kept in long-term memory (e.g., the location of a car in a parking lot after driving out of the parking lot).

**Figure 2:**
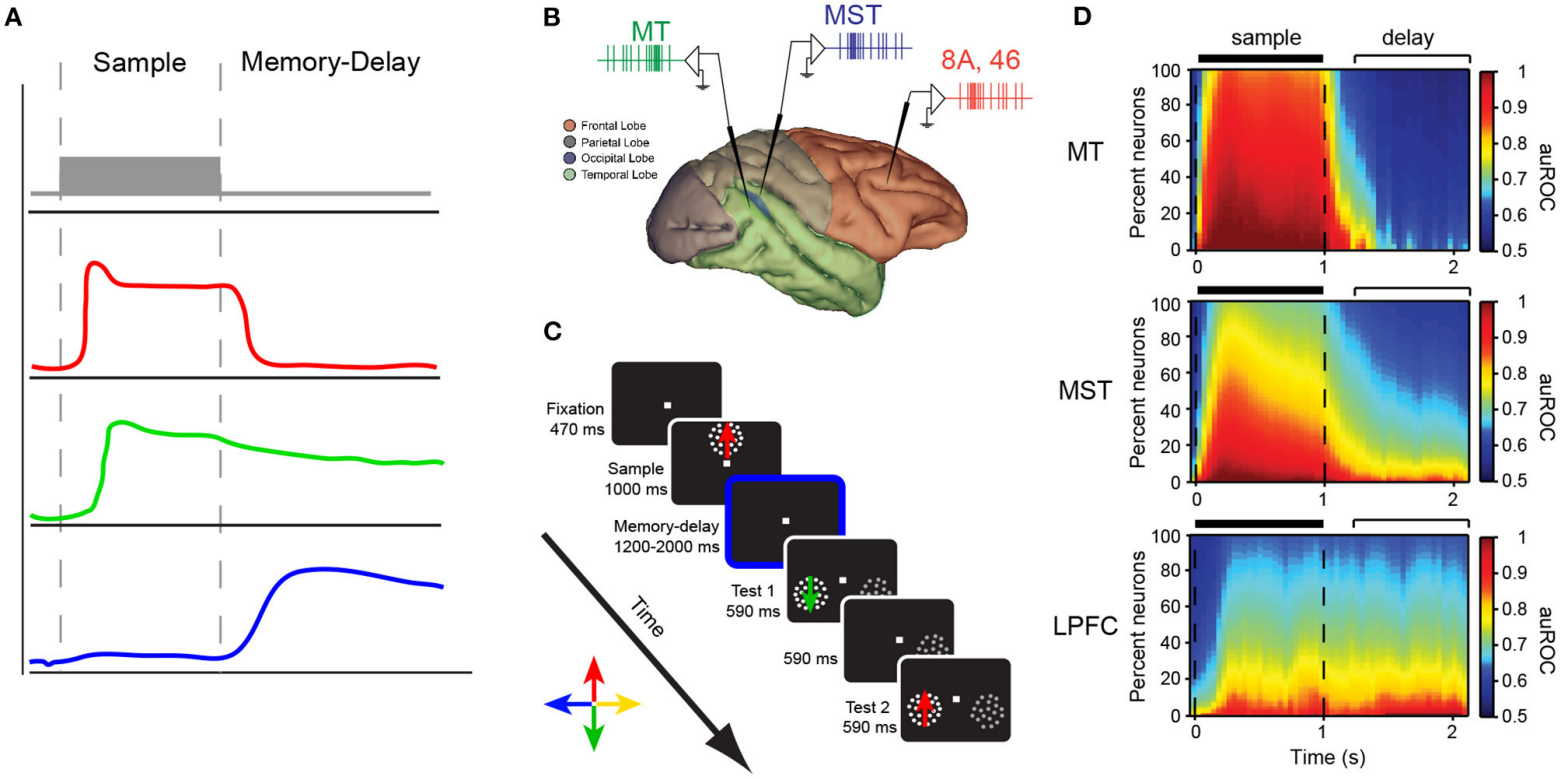
**(A)** Different response profiles of neurons in the LPFC of macaque monkeys during the sample and delay periods of a delayed response task. **(B)** Recording locations in the study of Mendoza-Halliday et al. (2014). MT (green), MST (blue), LPFC areas 8A/46 (red) during a match-to-sample task for motion direction **(C)**. **(D)** The proportion of neurons showing encoding of motion direction during the sample and delay period of the task in the three areas. The color scale represents the strength of direction selectivity quantified by the area under the receiver operating characteristic curve (auROC).

Fuster and Alexander also reported in their seminal work that a number of neurons in the LPFC were activated during the cue period of the delayed response task, whereas others were active only when the cue stimulus disappears. They suggested that the activity during the cue period may be related to attention since many neurons did not show selectivity for the position of the cue (Fuster and Alexander, 1971). Importantly, the fact that a group of neurons show activity exclusively during the delay period (mnemonic cells) suggests that, at the level of individual neurons, the neural correlates of working memory can be dissociated from those of visual perception (Figure 2A).

After this initial report, several studies have reported that persistent firing representing the contents of working memory can also be found in association areas of the frontal, parietal (Andersen et al., 1985), and temporal lobes (Mikami and Kubota, 1980; Fuster and Jervey, 1981); for a review see (Leavitt et al., 2017a). These findings sparked the debate on what the role of association areas outside the LPFC in WM coding is (Riley and Constantinidis, 2015). This question remains mostly unanswered but something that is common to studies in the PFC and posterior association cortices is the existence of neurons that represent information during different task periods. Thus, no matter where persistent firing has been reported, neurons showing selectivity for a visual cue are not necessarily the same as neurons showing persistent firing when a representation of a cue is held in working memory. The latter could be interpreted as evidence in favor of the hypothesis that the substrates for perception and working memory are at least partially segregated within areas such as the LPFC.

One study has reported that the proportion of neurons encoding information during the cue and delay period of a delayed match-to-sample task changes as one moves along the hierarchy of visual processing from area MT (neurons almost exclusively encode during the sample period) to MST (neurons predominantly encode information during the sample period but a proportion of cells also encode information during the delay period) to LPFC (a similar proportion of neurons encode information during the sample and delayed period) (see Figures 2B–D) (Mendoza-Halliday et al., 2014). Bisley et al. (2001) reported that microstimulation of area MT during the encoding stage of a working memory task for motion direction biased the neural response to direction but stimulation during the delay period did not. The latter supports the hypothesis that although sensory areas are recruited during visual processing and perception, which is require for encoding information during working memory tasks, they may play a lesser role in maintaining working memory representations. These results match the pattern revealed by lesion studies with neurons in the posterior early sensory and association areas encoding predominantly perceptual information and neurons in the PFC encoding mnemonic signals (Figures 2B–D). One may also conclude that a population of neurons in areas such as LPFC seem to encode information about the cue during all task periods (Mendoza-Halliday and Martinez-Trujillo, 2017).

Although we, as most researchers, discuss independent properties of various brain regions, it is important to expand beyond the local-circuit model and recognize the impact that cortical—cortical connections have in generating persistent activity. In 1998, Chafee and Goldman-Rakic made the observation that patterns of neuronal activity in the dorsolateral prefrontal cortex and parietal area LIP/7a were remarkably similar including their spatial tuning and ability to generate persistent activity (Chafee and Goldman-Rakic, 1998). They later demonstrated, using cortical cooling, that WM memory related activity in both regions were dependent on shared reciprocal activity (Chafee and Goldman-Rakic, 2000). Synchronized activity between PFC and PPC underlying working memory has since been substantiated (Salazar et al., 2012). The prefrontal and parietal cortices thus represent two regions in which persistent activity is frequently observed but the role of their reciprocal connections is still debated (Christophel et al., 2017; Constantinidis et al., 2018).

To explore the function of these prefrontal- parietal connections, Murray et al. (2017) developed a computational model of two bidirectionally connected modules that biophysically represented local networks of PFC and PPC. This model shows that PPC functions in a weak attractor state and transiently encodes the stimulus and propagates this sensory signal to PFC. Although both maintain the WM representation after stimulus offset, the attractor state is stronger in PFC module, allowing for robustness against distractors. Feedback projections from PFC can additionally switch PPC neurons back to encoding target stimuli after distractor presentation. Therefore, in this model, persistent activity was supported by both local and long-range network connections.

Synchronized activity was also identified between area MT and LPFC through observations of phase- coherent local field potential oscillations during a motion direction match to sample task. This observation suggests that persistent activity in LPFC modulates synaptic activity in MT, again showing a top-down mechanism by which memory signals in LPFC influence stimulus processing (Mendoza-Halliday et al., 2014).

Regarding the neural correlates of visual perception, there is a large body of literature starting as early as when single cell recording techniques became popular (Hubel, 1957). Early studies of Hubel and Wiesel demonstrated that neurons in the monkey primary visual cortex (V1) encode the features of sensory stimuli shown inside their receptive field (RF) (Hubel and Wiesel, 1968). Later studies discovered similar selectivity in other brain areas of both the dorsal and ventral visual pathways (Mikami et al., 1986). The selectivity for features and their conjunction becomes more complex in areas downstream from V1 (e.g., linear motion in MT and complex optic flow motion in MST, or color and orientation selectivity in V4 and face selectivity in IT) (Felleman and Van Essen, 1991). However, most of these studies focused on the specific role of brain areas in conscious visual perception rather than in the distinction between perception and mnemonic processes. For example, lesions of area V1 leaves subjects cortically blind; however, lesioned individuals may show some residual vision or blindsight, likely suggesting that some perception can happen without V1 (reviewed in Leopold, 2012). Nevertheless, many agree that visual perception is deeply impaired after V1 lesions, suggesting that V1 is a bottleneck for visual signals entering higher level areas of the visual pathways (Leopold, 2012).

Remarkably, selective deficits in motion perception without affecting contrast thresholds can be observed after lesions of area MT (Newsome and Pare, 1988). Area MT contains a high proportion of direction selective neurons that receive inputs from direction selective neurons in area V1 (Born and Bradley, 2005). These observations suggests that V1 is not sufficient for motion perception but necessitates area MT. This hypothesis has been supported by reports of electrical microstimulation in area MT neurons, biasing motion perception (Salzman and Newsome, 1994). On the ventral pathways, damage to areas of the temporal lobe, such as the fusiform face area, leads to prosopagnosia: a selective deficit in face perception (Barton, 2003). Cells selective for faces have been extensively reported in the macaque inferiortemporal cortex (Perrett et al., 1984; Freiwald and Tsao, 2012). One influential study used visual rivalry, a phenomenon in which two different images are presented separately to each eye, the subject experiences alternating percepts of each image and periods of fusion of the two images. Single neuron activity is reported to more accurately reflect the percept downstream from area V1 (Leopold and Logothetis, 1996). The latter suggests that although V1 activity is essential to perception, the phenomenology that triggers perceptual awareness may occur or at least be triggered in downstream areas such as MT or MST, where neurons selective for the perceived features exist.

A central question to this review is whether the neural substrates that support visual perception and those that support working memory are the same or different. From the previous sections we may conclude that: (1) there is a set of areas in which neurons represent visual attributes such as motion (Duffy and Wurtz, 1991) and complex shapes (Rolls, 1984) during both perception and working memory tasks (Miller et al., 1991; Mendoza-Halliday et al., 2014), (2) there is a set of areas where neurons encode perceptual but not mnemonic representations of visual attributes, mainly early areas in the hierarchy of visual processing (i.e., V1 to MT in the dorsal pathway, and V1 to V4 in the ventral pathway), and (3) the relative proportion of neurons showing selectivity for perceptual and mnemonic visual attributes changes along the hierarchy of visual processing (i.e., the proportion of cells encoding mnemonic relative to perceptual representations is lower in MST than in LPFC), and (4) there are different subpopulations of neurons encoding perceptual and mnemonic representations in association areas, as well as a subpopulation of neurons that encode both types of representations.

### 2.3. Coding of Perceptual and Working Memory Representations by Subpopulations of Neurons Within Brain Areas

**Figure 3:**
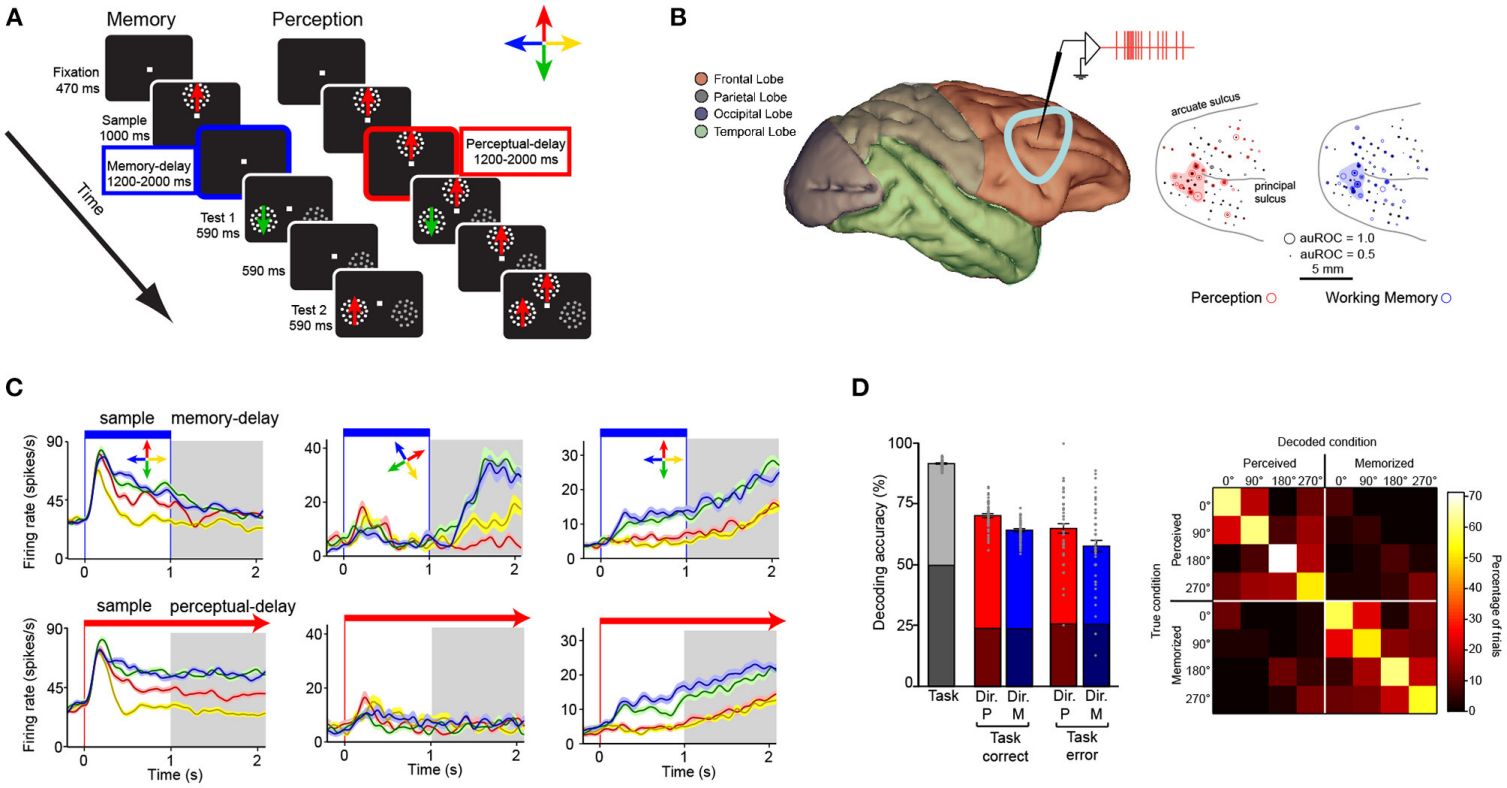
Encoding of perceptual and working memory representations by subpopulations of neurons within the LPFC. **(A)** Memory and perception tasks. Panels illustrate stimuli and monitor setup. Animals fixate a dot at the center of a computer screen and press a button. Then a sample Random Dot Pattern (RDP) appears moving in one of four directions. In the memory task (left) the sample disappears after 1,000 ms. A delay period of 1,200 to 2,000 ms then occurs in which only the fixation point is on the screen. At the end of the delay period two patterns, a test RDP moving in either the same or different direction as the sample, and a distracter RDP that contains dots moving in random directions are presented. The animal releases a button if the test matches the direction of the sample or waits until the test disappears, and a second test RDP is presented after a 590-ms delay period. During the perceptual (right) task the sample RDP does not disappear but stays on during the duration of the trial. **(B)** Recording locations in the LPFC. The dots indicate the location of units with selectivity during the memory (blue) and perceptual (red) tasks. **(C)** Firing rate (y axis) over time (x axis) for three example neurons (left, center, and right columns) during the working memory task (top row) and perceptual task (bottom row). The task periods are indicated on top. **(D)** left bar graph: Accuracy of a linear classifier to decode, from the population of recorded neurons, the task (working memory vs. perceptual tasks, gray bar), the direction of the stimulus in each task in trials with correct and incorrect task decoding (red and blue bars). Right panel: confusion matrix for the classification of perceived and memorized direction corresponding to the correct trials.

The exclusive role of the PFC and association cortices in working memory coding has recently been put into question (Pasternak and Greenlee, 2005; Christophel et al., 2017; Scimeca et al., 2018). Some studies have proposed that neurons in sensory areas such as V1 and V4 encode working memory representations (Pasternak and Greenlee, 2005; Tong and Pratte, 2012). One argument in favor of this idea is that single neurons and neuronal populations in early sensory areas contain precise maps of visual attributes (Hubel and Wiesel, 1968; Albright, 1984; Born and Bradley, 2005). Thus, these populations must be recruited for perceiving such attributes accurately (Ester et al., 2013). However, encoding of visual attributes by single neurons and populations does not exclusively occur in early sensory areas such as V1, MT, and V4 but also occurs in downstream association areas where the neural correlates of working memory have been isolated. One example is coding of linear motion direction, which has been found not only in MT, but also in MST and LPFC (Bisley et al., 2004; Zaksas and Pasternak, 2006; Mendoza-Halliday et al., 2014; Mendoza-Halliday and Martinez-Trujillo, 2017), as well as in areas such as the Lateral Intraparietal (LIP) area (Freedman and Assad, 2006). Another example is encoding of color which has been reported not only in area V4, but also in the LPFC (Schwedhelm et al., 2020). Something to point out is that feature-selective neurons in the LPFC do not exhibit the retinotopic or feature-topic organization observed in early sensory areas (see Figure 3B; Mendoza-Halliday and Martinez-Trujillo, 2017). Thus, human studies using functional imaging techniques or EEG/MEG, that pool activity over cubic millimeters of cortical tissue, may underestimate selectivity for individual features or locations.

One important detail we have already mentioned is that feature selectivity in association areas does not only occur during delayed response tasks, but also during perceptual tasks when a stimulus remains visible (Mendoza-Halliday and Martinez-Trujillo, 2017). Interestingly, single unit responses to the same visual attribute become more correlated with behavioral outcomes as one advances downstream from V1 in the hierarchy of visual processing, for example from MST to LPFC (Freedman et al., 2001; Freedman and Assad, 2006; Mendoza-Halliday et al., 2014). Thus, association areas are equipped with “copies” of perceptual representations likely inherited from upstream areas, as well as with mnemonic representations that may emerge as a result of local processing. Unlike in visual areas, such “copies” are sensitive to the statistics of the environment and can form categories within a single feature dimension (Freedman et al., 2001).

Indeed, association areas in the frontal lobe such as the LPFC (around the posterior third of the principal sulcus) contain neurons that encode motion direction during a delayed match-to-sample task as well as neurons that encode memory representations of the same motion direction (Mendoza-Halliday and Martinez-Trujillo, 2017) (Figures 3A,B). A study found that about 1/3 of the neurons encoded perceptual representations of motion direction but not mnemonic representations, another 1/3 encoded mnemonic representations but not perceptual representations, and another 1/3 encoded a mix of both perceptual and mnemonic representations (Mendoza-Halliday and Martinez-Trujillo, 2017). Importantly, mnemonic cells are selective for motion direction only during the delay period and not during the visual presentation of the same motion direction (Figure 3C middle panel). Perceptual cells show the opposite pattern. Perceptual and mnemonic cells show a concentration within the posterior end of the principal sulcus and were also found to be spread within area 9/46 but without any apparent clustering by the type of representation (perceptual or mnemonic) or the feature they encode (Figure 3B). The latter deviates from observations in early sensory areas such as MT where neurons are topographically organized according to their RF location and motion direction they encode (Born and Bradley, 2005). As mentioned before, exploring the fine granulated functional architecture of the LPFC using BOLD signal measurement or EEG/MEG with spatial resolution of millimeters may cause an under estimation of feature selectivity or selectivity for perceptual and mnemonic representations.

The segregation of the different populations (perceptual and mnemonic) within LPFC allows a linear decoder to use single neuron activity to estimate whether a direction of motion is held in working memory or is visually presented (perception-memory decoder) as well as which direction is perceived or memorized (direction decoder) (Figure 3D). This indicates that perceptual and mnemonic signals as well as the features they encode can be discriminated, with reasonable accuracy, from the activity of neurons within the LPFC circuitry.

The existence of subpopulations of perceptual and mnemonic neurons within the LPFC circuitry may be considered as evidence in favor of separate substrates for perception and working memory “concentrated” within a single brain area microcircuit. One potential functional relevance of such a concentration is that a “read-out” of the population activity in the LPFC can provide a substrate for rapidly “identifying” the nature of the representation—perceptual or mnemonic—as well as its content. In the language of dynamical systems, the different activity profiles during the perceptual and mnemonic states could serve as attractors for corresponding cognitive states respectively (Wimmer et al., 2014). Interestingly, in patients with schizophrenia that lose the ability to differentiate between perceptual and mental representations (e.g., during hallucinations and delusions), abnormal patterns of activity are commonly reported in areas such as the LPFC (Callicott et al., 2000). Working memory deficits are also common in patients with schizophrenia and abnormal LPFC activity is consistently reported (Glahn et al., 2005; Forbes et al., 2009). In favor of this hypothesis, we have recently reported that systemic administration of ketamine, a drug often used to model symptoms of schizophrenia, modulates the activity landscape in the LPFC of macaques. In this experiment, ketamine drastically reduced performance during a working memory task by destroying the tuning of prefrontal neuron delay activity for remembered locations but had no effect on a perceptual control version of the same task (Roussy et al., 2021).

Another possible functional relevance to the coexistence of perceptual and mnemonic signals in the LPFC, is that information transfer from perceptual to mnemonic neurons can happen locally through short range connections within the area microcircuit, without the need for transfer through long range connections (e.g., perceptual neurons in MST transferring information about the cue to mnemonic cells in LPFC). For example, during delayed matched-to-sample tasks, a read-out from sensory areas can be “loaded” into the perceptual cells and transferred “internally” to mnemonic cells that will “maintain” the representation via persistent firing. The role of perceptual and mnemonic cells in the generation of feedback signals that influence processing in early sensory areas is not clear. One study has documented synchrony between spikes in LPFC and local field potentials (LFPs) in MT during the delay period of a memory task (Mendoza-Halliday et al., 2014). Other studies have documented that microstimulation of areas such as the Frontal Eye Fields (FEF), posterior to LPFC, produces a modulation of responses in area V4 (Moore and Armstrong, 2003). Thus, it is possible that perceptual and mnemonic cells in LPFC play a critical role in modulating the activity of neurons in early visual areas during tasks that require attention either to sensory (perceptual) or mnemonic representations.

Finally, a concentration of neurons holding different representations of space, objects, and their attributes within a relatively small brain volume may facilitate the implementation of other cognitive operations such as attention. The predominant hypothesis of how attention is implemented is through competition via inhibitory interactions between neurons encoding representations of targets and distracters (Reynolds et al., 1999). Studies have reported evidence that the strength of such competition increases in association areas downstream from V1 (Buffalo et al., 2010; Lennert and Martinez-Trujillo, 2013). The strength of the competition also increases when targets and distracters become closer in space (Treue and Martínez Trujillo, 1999). Interestingly, association areas in the PFC possess spatial representations of the entire visual field, which may allow implementing competition between neurons representing targets and distracters in opposite hemifields via short range inhibitory connections within a local circuitry (Lennert and Martinez-Trujillo, 2013; Duong et al., 2019). Such operations could be more difficult to implement through short range projections between neurons in areas such as V1 or MT, where neurons represent stimuli in the opposite hemifield (Born and Bradley, 2005). Additionally, for the particular case of V1, with a large surface area, short range connections may be insufficient to implement operations when targets and distracters are far apart but still within the same hemifield. The latter may suggest the reduction in surface area from early visual areas relative to areas downstream facilitates interactions between neurons encoding different representations via short range connections.

### 2.4. Cortical Architectures for Perceptual and Mnemonic Coding

The primate cerebral cortex is not homogenous. Cortical architecture varies between early sensory and association areas in terms of thickness of cortical layers (Yang et al., 2018), neuronal densities (Collins et al., 2010), and proportion of different interneuron types (Torres-Gomez et al., 2020). The latter has been related to the ability of some local microcircuits to generate persistent firing in the absence of sensory stimulation (Leavitt et al., 2017a; Torres-Gomez et al., 2020). Indeed, the neural basis of persistent firing has been linked to the existence of recurrent connections between pyramidal cells within a local area circuitry (Goldman-Rakic, 1995). Empirical evidence shows more numerous excitatory synapses between pyramidal cells as well as differences in the distribution of long time constant NMDA receptors relative to short time constant AMPA receptors in the LPFC compared to the early visual cortex (Wang, 1999; Gonza'lez-Burgos et al., 2000; Zaitsev et al., 2012; Yang et al., 2018). These differences in excitatory synapse numbers and glutamate receptor types may explain the larger integration times found in association and executive areas of the visual processing hierarchy relative to sensory areas (Murray et al., 2014) and the ability of the former set of areas to encode working memory representations.

**Figure 4:**
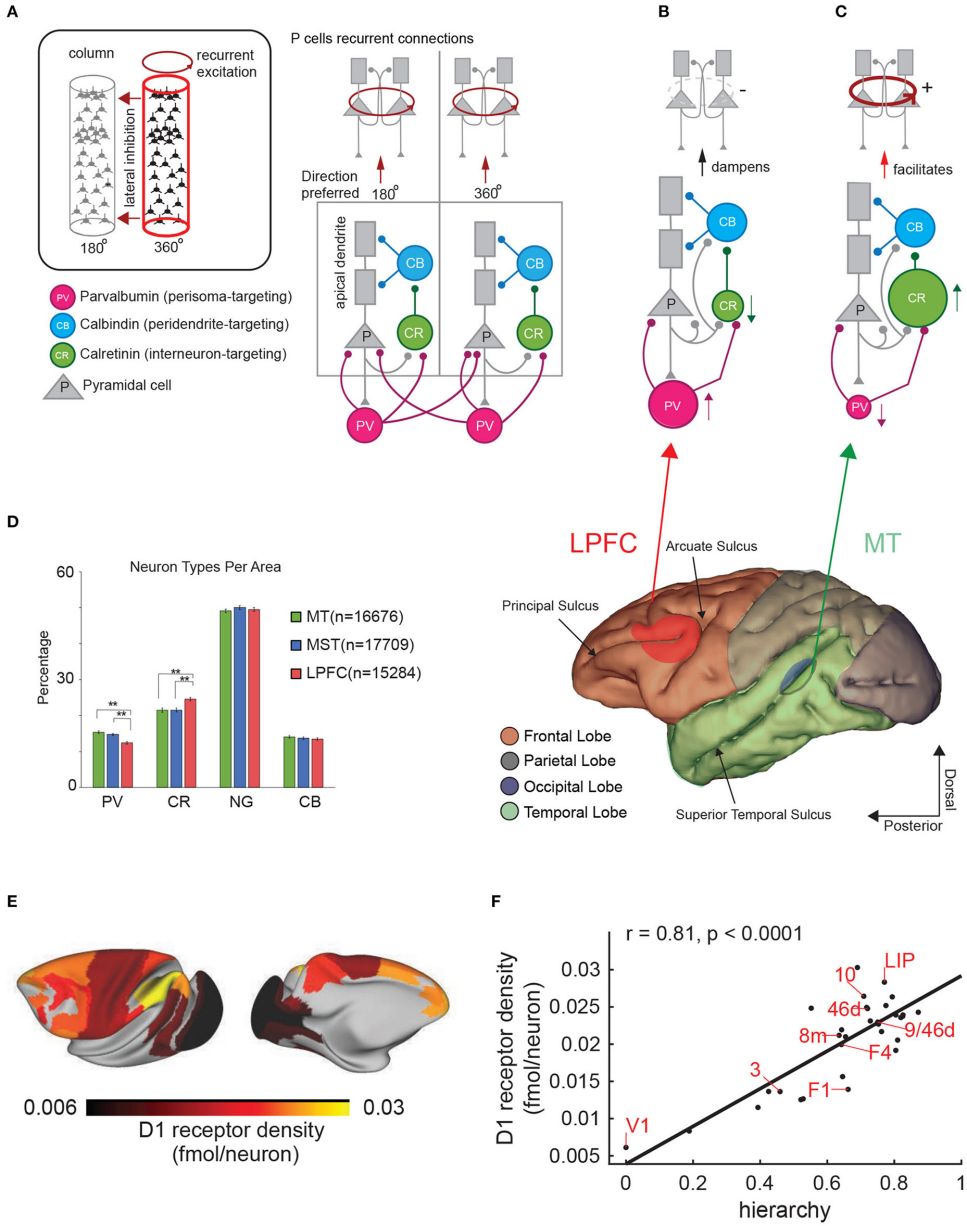
Cortical architectures for perception and working memory. **(A)** Diagram showing the structure of two nearby cortical columns and the four main cell types (see inset). Observe pyramidal cells have at least two distinct compartments, the apical (distal) dendrites (gray rectangles) and the cell body. **(B,C)** different architectures based on the proportion of CR and PV interneurons and the ability to produce persistent firing. Lower panel shows a side view of the macaque brain and the different lobes in different colors. **(D)** Percentages of the 4 main cell types in areas MT, MST, and the LPFC (from Torres-Gomez et al., 2020). Distribution of Dopamine D1 receptors in the macaque brain. The color scale indicates the receptor density. **(F)** Correlation between position of a brain area in the hierarchy of visual processing and D1 receptor density. Each data point represents a brain area. The correlation coefficient and associated *p-*value are indicated courtesy of Froudist-Walsh et al. (2020). **(E)** D1 receptor density across the macaque cortex.

More recently, a larger proportion of interneurons that disinhibit pyramidal cells (e.g., calretinin positive (CR) cells) relative to interneurons that directly inhibit pyramidal cell firing (e.g., parvalbumin (PV) positive cells) have been reported in the LPFC compared to early visual areas like MT (Torres-Gomez et al., 2020). Wang has elaborated on a model that incorporates different cell types within the LPFC circuitry such as the calretinin positive (CR, sometimes identified as functionally similar to vasointestinal peptide (VIP)-expressing neurons in mice) and the calbindin positive neuron (CB, sometimes identified as functionally similar to somatostatin (SST)-expressing neurons in mice) (Wang et al., 2004; Wang, 2009). CR cells receive inputs from pyramidal cells and inhibit CB cells. The CB cells inhibit inputs into the dendrites of pyramidal cells (Figure 4A). Thus, an increase in the number or activation strength of CR neurons or their synapses onto CB cells would have a positive impact on the activation of the pyramidal cells (Figure 4B). A decrease in CR numbers or synaptic strength on their targets may have the opposite effect (Figure 4C). On the other hand, for PV neurons, an increase in their proportion or relative synaptic strength would increase the inhibition of pyramidal cells. A high ratio of CR to PV cells in LPFC relative to sensory areas may favor the emergence of persistent firing encoding working memory via facilitation of recurrent excitatory dynamics amongst pyramidal cells (Torres-Gomez et al., 2020) (Figure 4D). A low ratio of CR to PV cells (e.g., a relatively high proportion of PV cells or synaptic strength onto their target pyramidal cells) may cause strong inhibition of pyramidal cell firing and dampening of recurrent excitatory dynamics (perceptual encoding).

Supporting the idea that cortical architectures differ in their interneuron type proportions, a recent study has compared transcriptomic profiles of different neuronal types [PV, SST, VIP, and LAMP5 (Lysosome associated membrane protein 5 expressing interneurons)] in areas V1 and PFC of different species of primates (common marmosets, rhesus macaques, and humans). SST and PV originate from the Medial Ganglionic Eminence (MGE), while the VIP and LAMP5 originate from the Caudal Ganglionic Eminence (CGE). Neurons originating from the MGE tend to be more numerous in the deep layers while those originating in the CGE tend to be more numerous in the superficial layers. The study found that whereas PV and SST cells are more abundant in area V1, VIP, and LAMP5 are relatively more abundant in PFC. These differences may be due to the expansion of superficial (supragranular) cortical layers in primate association cortices, better documented in LPFC (Arnsten et al., 2012). Interestingly, such differences in the proportion of interneuron types were not found in the mouse (Krienen et al., 2020; but see Kim et al., 2017). This suggests that gradients of interneuron types may have become pronounced in primate neocortex, which is compatible with studies reporting a larger proportion of interneurons in primates relative to rodents (Džaja et al., 2014), as well as a larger proportion of CR cells in LPFC relative to sensory areas (Torres-Gomez et al., 2020).

One issue that remains unclear is why areas such as MST, where neurons show persistent firing during working memory tasks, do not show the same increase in the ratio of CR to PV neurons observed in the LPFC. There may be two possible explanations for this result. First, that persistent firing in areas such as MST is not intrinsic to the area circuitry and needs strong feedback signals from LPFC. Second, it is possible that the differences in CR interneurons proportion described in previous studies (Torres-Gomez et al., 2020) is not directly related to the ability to produce persistent firing, but to the ability of a local area circuitry to make persistent firing encoding working memory representations less disrupted by incoming distracting sensory signals (e.g., sensory signals unrelated to the representation held in working memory but co-occurring during the period of memory maintenance). In favor of the latter explanation, inactivation of the LPFC, where CR interneurons are abundant, increases distracter interference during working memory tasks and activity in LPFC is less disrupted by incoming distracting signals than in areas such as LIP (Suzuki and Gottlieb, 2013).

Wang and Yang have proposed a model circuit motif composed of the same cell types referred to earlier (pyramidal, CB, CR, and PV). Here the dendrite targeting CB neurons can regulate the flow of signals into dendritic trees. These neurons are controlled by CR interneurons. An increase in these cell type proportions and their control by cognitive signals encoding the behavioral relevance of stimuli in the environment in areas like LPFC where the filtering of distracter signals is particularly strong (Lennert and Martinez-Trujillo, 2011), may allow flexible “gating” of inputs into a pyramidal cell network. An increase in the proportion of SST neurons, a putative functional homolog of CB neurons in primates, has been reported in association areas of the mouse neocortex (Kim et al., 2017). One issue that remains unclear is how the gating of sensory inputs from upstream areas interplay with the gating of recurrent excitatory inputs from neighboring cells within the area. Further exploration will clarify apparent contradictions between the aforementioned hypotheses regarding cell type gradients.

Another difference between early sensory and association cortices concerns the distribution of receptors for neuromodulators that have been classically involved in working memory functions (Brozoski et al., 1979). Froudist-Walsh and coworkers have recently shown that receptors for neuromodulators that regulates working memory function such as the dopamine D1 receptor (D1R) (Williams and Goldman-Rakic, 1995) has an unequal distribution in the macaque cerebral cortex (Froudist-Walsh et al., 2020) (Figures 4E,F). D1 dopamine receptors action have been associated with the ability to filter distracter stimuli (Jacob et al., 2016). The concentration of D1 receptors increases along the hierarchy of visual processing reaching their maximal concentration in the parietal and prefrontal cortices. Froudist-Walsh and coworkers elaborated on a computational model in which release of dopamine favors persistent firing and resilience to distracters in association areas via its action on D1 receptors. Insufficient or excessive dopamine release on the other hand, makes persistent firing less robust to distracter interference (Froudist-Walsh et al., 2020). One relevant detail is that the model makes the prediction that dopamine increases the synaptic strength of the inhibition to the apical dendrites of pyramidal cells. Because recurrent excitatory connections between pyramidal cells target the soma and proximal dendrites, which are NMDA dependent and facilitated by D1R, and inhibitory connections from calbindin-expressing interneurons target the apical dendrites and are also facilitated by D1R, the next effect for dopamine release is to facilitate persistent firing via recurrent excitation. Additional details of this model can be found in Froudist-Walsh et al. (2020).

Despite an accumulating body of evidence in favor of different cortical architectures that support perception and working memory, several issues remain unexplained. For example, studies have reported that a noticeable proportion of neurons in areas of the LPFC encode perceptual but not mnemonic representations (see Figure 3; Mendoza-Halliday and Martinez-Trujillo, 2017). Here one may conceive the possibility that the LPFC microcircuitry is heterogeneous in composition and may contain features of both perceptual and mnemonic microcircuits. One may speculate that perceptual neurons inherit and “echo” the responses and selectivity from perceptual neurons in upstream visual areas (e.g., MT) via feed-forward inputs, processing these signals within circuits that do not include mnemonic neurons. During working memory, perceptual LPFC neurons then transfer such signals to mnemonic neurons, which are in turn capable of maintaining them via local recurrent excitatory networks that do not necessarily include perceptual neurons.

Is it possible the LPFC is a mosaic of perceptual and mnemonic cortical architectures that differ in basic features such as proportion of interneuron types, or the number of synapses that enable recurrent connections? If that were the case, one may conceive evolution of the neocortex produced such a hybrid architecture for a “purpose”: compressing information about the nature of a representation (perceptual or mnemonic) within a brain area. One possibility is that such architecture originates, at least partially, during migrations of interneurons from MGE (e.g., PV) and CGE (e.g., CR/VIP) that produce cortical columns of different composition in areas such as LPFC. It may also be shaped by patterns of inputs and activity during development. As we have proposed earlier, this hybrid architecture may facilitate computations and information transfer within local microcircuits in an efficient manner. On the other hand, it may also make the brain more vulnerable to disorders of perception/imagination when such a circuit undergoes certain deviations from typical development in early life, as can be seen in schizophrenia. This idea, however, needs to be tested experimentally.

An interesting question related to the possible existence of a hybrid architecture of perceptual or mnemonic “blocks” in the LPFC, is what the resolution of such blocks would be. Some studies have pointed out the existence of a non-retinotopic topography for mnemonic representations of visual space in the macaque monkey LPFC (Leavitt et al., 2017b). Another study found that neurons with the strongest selectivity for perceived and memorized motion directions were concentrated within a small subregion of LPFC near the posterior end of the principal sulcus (Mendoza-Halliday and Martinez-Trujillo, 2017). Moreover, a previous study has described a pattern of stripe-like areas in the LPFC that connects to the ipsilateral parietal cortex and the contralateral LPFC respectively (Goldman-Rakic and Schwartz, 1982). Could such a pattern be related to subregions of LPFC with perceptual and mnemonic architectures such as the ones illustrated in Figures 4B,C? One possibility is that neurons in perceptual blocks receive projections from the parietal cortex, while neurons in the mnemonic blocks receive projections from perceptual blocks within the same hemisphere and contralateral blocks in the opposite hemisphere. The latter may allow manipulation of spatial information in working memory (e.g., interhemispheric transfer of information; Brincat et al., 2021). However, this proposal remains speculative and future studies must clarify this issue. With the advent of modern techniques for high-yield electrophysiological recordings and 2-photon imaging of neuronal activity using calcium indicators (Yang and Yuste, 2017), it may be possible to test some of the hypotheses mentioned or proposed here.

### 2.5. The Case for Overlapping Substrates of Visual Working Memory and Perception

With the advent of modern functional imaging, it has been possible to measure Blood Oxygenation Level Dependent (BOLD) signals in humans performing perceptual and working memory tasks. One common finding is that it is possible to decode the contents of working memory from BOLD signals in early visual areas (V1-V4) (Tong and Pratte, 2012). Yet, electrophysiological studies in monkeys find little evidence of persistent firing of action potential by single neurons (see Leavitt et al., 2017a for a review). These functional imaging findings have been the motivation of a popular hypothesis that proposes early sensory areas are recruited, and may be necessary, for the maintenance of working memory representations (Postle, 2006; Ester et al., 2013; Scimeca et al., 2018). This hypothesis is known as the “sensory recruitment” hypothesis, and has been a matter of debate amongst neuroscientists investigating the topic (Scimeca et al., 2018). At first glance, the sensory recruitment hypothesis does not fully match the results of electrophysiological and lesion studies in non-human primates we have reviewed above. Below, we consider a few explanations for this mismatch.

Boynton (2011) outlines several hypotheses to understand the identified discrepancies between single neuron electrophysiology and fMRI findings. The first outlines that the BOLD signal more closely represents local field potential activity rather than spiking activity. It is possible that sensory areas are not recruited during working memory maintenance and the results of fMRI studies reflect feedback signals from higher-order association areas into early sensory areas. Such signals would increase synaptic activity and oxygen consumption in early visual cortex in a retinotopic or feature-topic fashion, which is sufficient to produce BOLD signals that provide information about remembered locations/features, but insufficient to significantly evoke action potentials from single neurons. In favor of this hypothesis, at least one study in monkeys has reported the direction of a stimulus held in working memory can be decoded from LFP signals recorded in area MT but cannot be decoded from spiking activity of neurons within the area (Mendoza-Halliday et al., 2014). Indeed, previous studies have shown that in certain experimental conditions, it is possible to dissociate between the inputs into a cell and the spiking outputs: BOLD signals are better correlated with LFP signals (as a measure of synaptic inputs) than with spikes (Logothetis and Wandell, 2004). The feedback signals into early visual cortex would help implement top-down attention, facilitating or prioritizing the processing of incoming stimuli that match the features or locations held in working memory (Mendoza et al., 2011). Such effects are commonly found in visual search paradigms (Bichot et al., 2019) and have been interpreted as top-down modulation of neuronal activity in early visual areas by attentional templates (working memory signals) originating in executive control areas of the parietal and PFC.

One issue that also needs clarification is why classification accuracy during working memory tasks is poorer using BOLD signals recorded in parietal areas and the LPFC compared to early visual cortex (e.g., V1, V4, MT) (Bettencourt and Xu, 2016; Ester et al., 2016). One possible explanation is that the retinotopy of visual space is weaker in high-order association cortices, leading to reduced decoding performance for working memory using BOLD signals (Xu, 2017). Here, one may consider that decoding methods used in fMRI rely on the selectivity of voxels for remembered features or locations. Such voxels are usually isotropic and distributed in a way that map BOLD signals in the cortex homogeneously. Although a voxel in areas like V1 and MT may include neurons with similar selectivities (Born and Bradley, 2005), this is not the case in late association areas such as the LPFC, where retinotopic and feature-topic maps are not homogenous (Leavitt et al., 2017b) (see Figure 3).

Boynton (2011) also suggest that discrepancies are caused by differences in experimental design including the use of different species. The same research group is unlikely to study both macaques and humans and use both fMRI and single neuron recording techniques. Differences in experimental approach and design and interpretation of results could certainly contribute to the observed discrepancies. Another possible explanation is that humans differ from other primates such as macaque monkeys in the way in which working memory networks encode information in the brain. The recruitment of early sensory areas could be a feature of the human cortex that is not present in macaques and other species of monkeys. This hypothesis is difficult to test. We did not find any study in humans recording neuronal activity in early visual areas during working memory tasks. Methods such as fMRI, EEG, and MEG do not have sufficient spatial resolution to measure spikes in single neurons. They are most sensitive to transient changes in sensory inputs or behavioral states. Recordings of single neurons from areas such as V1 in human subjects during working memory tasks would clarify the issue. However, these experiments are difficult due to ethical constraints, and are exclusively performed in patients with clinically-implanted electrodes for epilepsy mapping, almost all of which do not target early visual areas. Although we cannot fully discard this hypothesis, it would assume that humans have undergone a major step in the evolution of working memory mechanisms and cortical architectures. Beside the expansion of the PFC and the more pronounced folding of the brain surface in humans, there is no evidence in favor of fundamental changes in circuitry between macaques and humans (Passingham and Wise, 2012). Future studies in humans may clarify this issue.

## 3. Conclusion

We conclude that the neural substrates of working memory and perception are segregated in the non-human primate neocortex. Neurons and neuronal populations in early visual areas mainly encode perceptual signals. In areas downstream, there are populations of neurons that encode both perceptual and working memory signals, with the relative proportion of neurons encoding the latter increasing from early association areas to the PFC. In the LPFC, the activity of neuronal populations can provide a neural substrate for the distinction between perceptual and mnemonic states via population activity profiles that can be translated into attractor landscapes. Changes in the architecture of microcircuits across the hierarchy of visual processing in terms of pyramidal cell morphology and connectivity, proportion of different interneuron types, and distribution of receptors (i.e., NMDA, AMPA, and dopaminergic) also reflect the changes in electrophysiological signals supporting perception and working memory. This suggests a parallel degree of heterogeneity between anatomy and physiology. Finally, the results from non-human primate studies do not match the proposition of a sensory recruitment hypothesis for working memory. The latter could be due to the heterogeneity of signal measurements and their interpretation across studies in humans and non-human primates, or to evolutionary changes in the mechanisms by which humans encode perceptual and working memory signals.

## Author Contributions

MR and JM-T contributed to the topic development, manuscript writing, and figure and table development. DM-H contributed to manuscript writing and figure development. All authors contributed to the article and approved the submitted version.

## Funding

This study was supported by NEURONEX-NSF/CIHR/DFG consortium, CIHR project grant to JM-T, CFI, NSERC Discovery grant to JM-T, and Autism Research Chair Government of Ontario to JM-T.

## Conflict of Interest

The authors declare that the research was conducted in the absence of any commercial or financial relationships that could be construed as a potential conflict of interest.

## Publisher's Note

All claims expressed in this article are solely those of the authors and do not necessarily represent those of their affiliated organizations, or those of the publisher, the editors and the reviewers. Any product that may be evaluated in this article, or claim that may be made by its manufacturer, is not guaranteed or endorsed by the publisher.

## References

[B1] Albright T. D. (1984). Direction and orientation selectivity of neurons in visual area MT of the macaque. J. Neurophysiol. 52, 1106–1130. 10.1152/jn.1984.52.6.1106 6520628

[B2] Andersen R. A. Essick G. K. Siegel R. M. (1985). Encoding of spatial location by posterior parietal neurons. Science 230, 456–458. 10.1126/science.4048942 4048942

[B3] Arnsten A. F. T. (2013). The neurobiology of thought: the groundbreaking discoveries of Patricia Goldman-Rakic 1937–2003. Cereb. Cortex 23, 2269–2281. 10.1093/cercor/bht195 23926115 PMC3767966

[B4] Arnsten A. F. T. Wang M. J. Paspalas C. D. (2012). Neuromodulation of thought: flexibilities and vulnerabilities in prefrontal cortical network synapses. Neuron 76, 223–239. 10.1016/j.neuron.2012.08.038 23040817 PMC3488343

[B5] Baddeley A. (2010). Working memory. Curr. Biol. 20, R136–R140. 10.1016/j.cub.2009.12.014 20178752

[B6] Barton J. J. (2003). Disorders of face perception and recognition. Neurol. Clin. 21, 521–548. 10.1016/s0733-8619(02)00106-8 12916490

[B7] Bauer R. H. Fuster J. M. (1976). Delayed-matching and delayed-response deficit from cooling dorsolateral prefrontal cortex in monkeys. J. Comp. Physiol. Psychol. 90, 293–302. 10.1037/h0087996 819472

[B8] Behrmann M. Moscovitch M. Winocur G. (1994). Intact visual imagery and impaired visual perception in a patient with visual agnosia. J. Exp. Psychol. Hum. Percept. Perform. 20, 1068–1087. 10.1037//0096-1523.20.5.1068 7964528

[B9] Beran M. J. Menzel C. R. Parrish A. E. Perdue B. M. Sayers K. Smith J. D.. (2016). Primate cognition: attention, episodic memory, prospective memory, self-control, and metacognition as examples of cognitive control in nonhuman primates. Wiley Interdiscipl. Rev. Cogn. Sci. 7, 294–316. 10.1002/wcs.1397 27284790 PMC5173379

[B10] Bettencourt K. C. Xu Y. (2016). Decoding the content of visual short-term memory under distraction in occipital and parietal areas. Nat. Neurosci. 19, 150–157. 10.1038/nn.4174 26595654 PMC4696876

[B11] Bianchi L. (1895). The functions of the frontal lobes. Brain 18, 497–522. 10.1093/brain/18.4.497

[B12] Bichot N. P. Xu R. Ghadooshahy A. Williams M. L. Desimone R. (2019). The role of prefrontal cortex in the control of feature attention in area V4. Nat. Commun. 10, 1–12. 10.1038/s41467-019-13761-7 31844117 PMC6915702

[B13] Bisley J. W. Zaksas D. Droll J. A. Pasternak T. (2004). Activity of neurons in cortical area MT during a memory for motion task. J. Neurophysiol. 9, 286–300. 10.1152/jn.00870.2003 14523065

[B14] Bisley J. W. Zaksas D. Pasternak T. (2001). Microstimulation of cortical area MT affects performance on a visual working memory task. J. Neurophysiol. 85, 187–196. 10.1152/jn.2001.85.1.187 11152719

[B15] Blum R. A. (1952). Effects of subtotal lesions of frontal granular cortex on delayed reaction in monkeys. AMA Arch. Neurol. Psychiatry 67, 375–386. 10.1001/archneurpsyc.1952.02320150108012 14894008

[B16] Born R. T. Bradley D. C. (2005). Structure and function of visual area MT. Annu. Rev. Neurosci. 28, 157–189. 10.1146/annurev.neuro.26.041002.131052 16022593

[B17] Boynton G. (2011). Spikes, BOLD, attention, and awareness: a comparison of electrophysiological and fMRI signals in V1. J. Vis. 11:12. 10.1167/11.5.12 22199162 PMC4124818

[B18] Brincat S. L. Donoghue J. A. Mahnke M. K. Kornblith S. Lundqvist M. Miller E. K. (2021). Interhemispheric transfer of working memories. Neuron 109, 1055–1066.e4. 10.1016/j.neuron.2021.01.016 33561399 PMC9134350

[B19] Brozoski T. J. Brown R. M. Rosvold H. E. Goldman P. S. (1979). Cognitive deficit caused by regional depletion of dopamine in prefrontal cortex of rhesus monkey. Science 205, 929–932. 10.1126/science.112679 112679

[B20] Buffalo E. A. Fries P. Landman R. Liang H. Desimone R. (2010). A backward progression of attentional effects in the ventral stream. Proc. Natl. Acad. Sci. U.S.A. 107, 361–365. 10.1073/pnas.0907658106 20007766 PMC2806732

[B21] Butters N. Pandya D. (1969). Retention of delayed-alternation: effect of selective lesions of sulcus principalis. Science 165, 1271–1273. 10.1126/science.165.3899.1271 4979528

[B22] Butters N. Pandya D. Sanders K. Dye P. (1971). Behavioral deficits in monkeys after selective lesions within the middle third of sulcus principalis. J. Comp. Physiol. Psychol. 76, 8–14. 10.1037/h0031037 4997740

[B23] Callicott J. H. Bertolino A. Mattay V. S. Langheim F. J. Duyn J. Coppola R.. (2000). Physiological dysfunction of the dorsolateral prefrontal cortex in schizophrenia revisited. Cereb. Cortex 10, 1078–1092. 10.1093/cercor/10.11.1078 11053229

[B24] Campbell R. J. Harlow H. F. (1945). Problem solution by monkeys following bilateral removal of the prefrontal areas. V. Spatial delayed reactions. J. Exp. Psychol. 35, 110–126. 10.1037/h0062913 18910258

[B25] Chafee M. V. Goldman-Rakic P. S. (1998). Matching patterns of activity in primate prefrontal area 8a and parietal area 7ip neurons during a spatial working memory task. J. Neurophysiol. 79,2919–2940. 9636098 10.1152/jn.1998.79.6.2919

[B26] Chafee M. V. Goldman-Rakic P. S. (2000). Inactivation of parietal and prefrontal cortex reveals interdependence of neural activity during memory-guided saccades. J. Neurophysiol. 83, 1550–1566. 10.1152/jn.2000.83.3.1550 10712479

[B27] Chalupa L. M. Werner J. S. (2003). The Visual Neurosciences. Cambridge, MA: MIT Press.

[B28] Charcot M. Bernard D. (1883). Un cas de suppression brusque et isole'e de la vision mentaledes signes et des objets (formes et couleurs). Prog. Med. 11,568–571.

[B29] Chow K. L. Blum J. S. Blum R. A. (1951). Effects of combined destruction of frontal and posterior associative areas in monkeys. J. Neurophysiol. 14, 59–71. 10.1152/jn.1951.14.1.59 14784874

[B30] Christophel T. B. Klink P. C. Spitzer B. Roelfsema P. R. Haynes J.-D. (2017). The distributed nature of working memory. Trends Cogn. Sci. 21, 111–124. 10.1016/j.tics.2016.12.007 28063661

[B31] Collins C. E. Airey D. C. Young N. A. Leitch D. B. Kaas J. H. (2010). Neuron densities vary across and within cortical areas in primates. Proc. Natl. Acad. Sci. U.S.A. 107, 15927–15932. 10.1073/pnas.1010356107 20798050 PMC2936588

[B32] Constantinidis C. Funahashi S. Lee D. Murray J. D. Qi X.-L. Wang M.. (2018). Persistent spiking activity underlies working memory. J. Neurosci. 38, 7020–7028. 10.1523/JNEUROSCI.2486-17.2018 30089641 PMC6083457

[B33] Croxson P. L. Kyriazis D. A. Baxter M. G. (2011). Cholinergic modulation of a specific memory function of prefrontal cortex. Nat. Neurosci. 14, 1510–1512. 10.1038/nn.2971 22057191 PMC3432567

[B34] Dijkstra N. Kok P. Fleming S. M. (2021). Perceptual reality monitoring: neural mechanisms dissociating imagination from reality. PsyArXiv. 10.31234/osf.io/zngeq 35122782

[B35] Duffy C. J. Wurtz R. H. (1991). Sensitivity of MST neurons to optic flow stimuli. I. A continuum of response selectivity to large-field stimuli. J. Neurophysiol. 65, 1329–1345. 10.1152/jn.1991.65.6.1329 1875243

[B36] Duong L. Leavitt M. Pieper F. Sachs A. Martinez-Trujillo J. (2019). A normalization circuit underlying coding of spatial attention in primate lateral prefrontal cortex. eNeuro 6:ENEURO.0301–18.2019. 10.1523/ENEURO.0301-18.2019 31001577 PMC6469883

[B37] Džaja D. Hladnik A. Bičanič I. Bakovič M. Petanjek Z. (2014). Neocortical calretinin neurons in primates: increase in proportion and microcircuitry structure. Front. Neuroanat. 8:103. 10.3389/fnana.2014.00103 25309344 PMC4174738

[B38] Eccles J. C. (1986). Mechanisms of long-term memory. J. Physiol. 81, 312–317. 3572825

[B39] Ester E. F. Anderson D. E. Serences J. T. Awh E. (2013). A neural measure of precision in visual working memory. J. Cogn. Neurosci. 25, 754–761. 10.1162/jocna00357 23469889 PMC4041615

[B40] Ester E. F. Rademaker R. L. Sprague T. C. (2016). How do visual and parietal cortex contribute to visual short-term memory? eNeuro 3:ENEURO.0041-16.2016. 10.1523/ENEURO.0041-16.2016 27294194 PMC4901147

[B41] Felleman D. J. Van Essen D. C. (1991). Distributed hierarchical processing in the primate cerebral cortex. Cereb. Cortex 1, 1–47. 10.1093/cercor/1.1.1-a 1822724

[B42] Finan J. (1942). Delayed response with pre-delay reinforcement in monkeys after the removal of the frontal lobes. Am. J. Psychol. 55, 202–214. 10.2307/1417079

[B43] Forbes N. F. Carrick L. A. McIntosh A. M. Lawrie S. M. (2009). Working memory in schizophrenia: a meta-analysis. Psychol. Med. 39, 889–905. 10.1017/S0033291708004558 18945379

[B44] Foster D. H. (2011). Color constancy. Vision Res. 51, 674–700. 10.1016/j.visres.2010.09.006 20849875

[B45] Freedman D. J. Assad J. A. (2006). Experience-dependent representation of visual categories in parietal cortex. Nature 443, 85–88. 10.1038/nature05078 16936716

[B46] Freedman D. J. Riesenhuber M. Poggio T. Miller E. K. (2001). Categorical representation of visual stimuli in the primate prefrontal cortex. Science 291, 312–316. 10.1126/science.291.5502.312 11209083

[B47] Freiwald W. Tsao D. (2012). Taking Apart the Neural Machinery of Face Processing. Oxford: Oxford University Press. 10.1093/oxfordhb/9780199559053.013.0036

[B48] Froudist-Walsh S. Bliss D. P. Ding X. Jankovic-Rapan L. Niu M. Knoblauch K.. (2020). A dopamine gradient controls access to distributed working memory in monkey cortex. bioRxiv 2020.09.07.286500. 10.1101/2020.09.07.286500 34536352 PMC8571070

[B49] Funahashi S. Bruce C. J. Goldman-Rakic P. S. (1993). Dorsolateral prefrontal lesions and oculomotor delayed-response performance: evidence for mnemonic “scotomas”. J. Neurosci. 13, 1479–1497. 10.1523/JNEUROSCI.13-04-01479.1993 8463830 PMC6576716

[B50] Fuster J. M. Alexander G. E. (1970). Delayed response deficit by cryogenic depression of frontal cortex. Brain Res. 20, 85–90. 10.1016/0006-8993(70)90156-3 4986430

[B51] Fuster J. M. Alexander G. E. (1971). Neuron activity related to short-term memory. Science 173, 652–654. 10.1126/science.173.3997.652 4998337

[B52] Fuster J. M. Bauer R. H. (1974). Visual short-term memory deficit from hypothermia of frontal cortex. Brain Res. 81, 393–400. 10.1016/0006-8993(74)90838-5 4434203

[B53] Fuster J. M. Jervey J. P. (1981). Inferotemporal neurons distinguish and retain behaviorally relevant features of visual stimuli. Science 212, 952–955. 10.1126/science.7233192 7233192

[B54] Glahn D. C. Ragland J. D. Abramoff A. Barrett J. Laird A. R. Bearden C. E.. (2005). Beyond hypofrontality: a quantitative meta-analysis of functional neuroimaging studies of working memory in schizophrenia. Hum. Brain Mapp. 25, 60–69. 10.1002/hbm.20138 15846819 PMC6871703

[B55] Goldman P. S. Rosvold H. E. (1970). Localization of function within the dorsolateral prefrontal cortex of the rhesus monkey. Exp. Neurol. 27, 291–304. 10.1016/0014-4886(70)90222-0 4987453

[B56] Goldman P. S. Rosvold H. E. Vest B. Galkin T. W. (1971). Analysis of the delayed-alternation deficit produced by dorsolateral prefrontal lesions in the rhesus monkey. J. Comp. Physiol. Psychol. 77, 212–220. 10.1037/h0031649 5000659

[B57] Goldman-Rakic P. S. (1995). Cellular basis of working memory. Neuron 14, 477–485. 10.1016/0896-6273(95)90304-6 7695894

[B58] Goldman-Rakic P. S. Schwartz M. L. (1982). Interdigitation of contralateral and ipsilateral columnar projections to frontal association cortex in primates. Science 216, 755–757. 10.1126/science.6177037 6177037

[B59] Gonza'lez-Burgos G. Barrionuevo G. Lewis D. A. (2000). Horizontal synaptic connections in monkey prefrontal cortex: an *in vitro* electrophysiological study. Cereb. Cortex 10, 82–92. 10.1093/cercor/10.1.82 10639398

[B60] Gross C. Weiskrantz L. (1962). Evidence for dissociation of impairment on auditory discrimination and delayed response following lateral frontal lesions in monkeys. Exp. Neurol. 5, 453–476. 10.1016/0014-4886(62)90057-2 13902195

[B61] Harlow H. F. Davis R. T. Settlage P. H. Meyer D. R. (1952). Analysis of frontal and posterior association syndromes in brain-damaged monkeys. J. Comp. Physiol. Psychol. 45, 419–429. 10.1037/h0056634 13000009

[B62] Hubel D. H. (1957). Tungsten microelectrode for recording from single units. Science 125, 549–550. 10.1126/science.125.3247.549 17793797

[B63] Hubel D. H. Wiesel T. N. (1968). Receptive fields and functional architecture of monkey striate cortex. J. Physiol. 195, 215–243. 10.1113/jphysiol.1968.sp008455 4966457 PMC1557912

[B64] Iba M. Sawaguchi T. (2003). Involvement of the dorsolateral prefrontal cortex of monkeys in visuospatial target selection. J. Neurophysiol. 89, 587–599. 10.1152/jn.00148.2002 12522204

[B65] Jacob S. N. Stalter M. Nieder A. (2016). Cell-type-specific modulation of targets and distractors by dopamine D1 receptors in primate prefrontal cortex. Nat. Commun. 7, 13218–13211. 10.1038/ncomms13218 27807366 PMC5095292

[B66] Jacobsen C. Wolfe J. Jackson T. A. (1935). An experimental analysis of the functions of the frontal association areas in primates. J. Nerv. Ment. Dis. 82, 1–14. 10.1097/00005053-193507000-00001

[B67] Jacobsen C. F. (1936). Studies of cerebral function in primates. I. The functions of the frontal association areas in monkeys. Comp. Psychol. Monogr. 13, 1–60.

[B68] Jacobsen C. F. Nissen H. W. (1937). Studies of cerebral function in primates. IV. The effects of frontal lobe lesions on the delayed alternation habit in monkeys. J. Comp. Psychol. 23, 101–112. 10.1037/h0056632

[B69] Kim Y. Yang G. R. Pradhan K. Venkataraju K. U. Bota M. Garc'ia Del Molino L. C.. (2017). Brain-wide maps reveal stereotyped cell-type-based cortical architecture and subcortical sexual dimorphism. Cell 171, 456–469. 10.1016/j.cell.2017.09.020 28985566 PMC5870827

[B70] Krienen F. M. Goldman M. Zhang Q. del Rosario R. C. H. Florio M. Machold R.. (2020). Innovations present in the primate interneuron repertoire. Nature 586, 262–269. 10.1038/s41586-020-2781-z 32999462 PMC7957574

[B71] Kubota K. Niki H. (1971). Prefrontal cortical unit activity and delayed alternation performance in monkeys. J. Neurophysiol. 34, 337–347. 10.1152/jn.1971.34.3.337 4997822

[B72] Leavitt M. L. Mendoza-Halliday D. Martinez-Trujillo J. C. (2017a). Sustained activity encoding working memories: not fully distributed. Trends Neurosci. 40, 328–346. 10.1016/j.tins.2017.04.004 28515011

[B73] Leavitt M. L. Pieper F. Sachs A. J. Martinez-Trujillo J. C. (2017b). A quadrantic bias in prefrontal representation of visual-mnemonic space. Cereb. Cortex 52, 1–17. 10.1093/cercor/bhx142 28605513

[B74] Lennert T. Martinez-Trujillo J. (2011). Strength of response suppression to distracter stimuli determines attentional-filtering performance in primate prefrontal neurons. Neuron 70, 141–152. 10.1016/j.neuron.2011.02.041 21482363

[B75] Lennert T. Martinez-Trujillo J. C. (2013). Prefrontal neurons of opposite spatial preference display distinct target selection dynamics. J. Neurosci. 33, 9520–9529. 10.1523/JNEUROSCI.5156-12.2013 23719818 PMC6618559

[B76] Leopold D. A. (2012). Primary visual cortex: awareness and blindsight. Annu. Rev. Neurosci. 35, 91–109. 10.1146/annurev-neuro-062111-150356 22715879 PMC3476047

[B77] Leopold D. A. Logothetis N. K. (1996). Activity changes in early visual cortex reflect monkeys' percepts during binocular rivalry. Nature 379, 549–553. 10.1038/379549a0 8596635

[B78] Logothetis N. K. Wandell B. A. (2004). Interpreting the BOLD signal. Annu. Rev. Physiol. 66, 735–769. 10.1146/annurev.physiol.66.082602.092845 14977420

[B79] Lundqvist M. Herman P. Miller E. K. (2018). Working memory: delay activity, yes! Persistent activity? Maybe not. J. Neurosci. 38, 7013–7019. 10.1523/JNEUROSCI.2485-17.2018 30089640 PMC6083456

[B80] Lundqvist M. Rose J. Herman P. Brincat S. L. Buschman T. J. Miller E. K. (2016). Gamma and beta bursts underlie working memory. Neuron 90, 152–164. 10.1016/j.neuron.2016.02.028 26996084 PMC5220584

[B81] Malmo R. B. (1942). Interference factors in delayed response in monkeys after removal of frontal lobes. J. Neurophysiol. 5, 295–308. 10.1152/jn.1942.5.4.295

[B82] Mendoza D. Schneiderman M. Kaul C. Martinez-Trujillo J. (2011). Combined effects of feature-based working memory and feature-based attention on the perception of visual motion direction. J. Vis. 11:11. 10.1167/11.1.11 21220539

[B83] Mendoza-Halliday D. Martinez-Trujillo J. C. (2017). Neuronal population coding of perceived and memorized visual features in the lateral prefrontal cortex. Nat. Commun. 8:15471. 10.1038/ncomms15471 28569756 PMC5461493

[B84] Mendoza-Halliday D. Torres S. Martinez-Trujillo J. C. (2014). Sharp emergence of feature- selective sustained activity along the dorsal visual pathway. Nat. Neurosci. 17, 1255–1262. 10.1038/nn.3785 25108910 PMC4978542

[B85] Mikami A. Kubota K. (1980). Inferotemporal neuron activities and color discrimination with delay. Brain Res. 182, 65–78. 10.1016/0006-8993(80)90830-6 6766080

[B86] Mikami A. Newsome W. T. Wurtz R. H. (1986). Motion selectivity in macaque visual cortex. I. Mechanisms of direction and speed selectivity in extrastriate area MT. J. Neurophysiol. 55, 1308–1327. 10.1152/jn.1986.55.6.1308 3016210

[B87] Miles R. C. Blomquist A. J. (1960). Frontal lesions and behavioral deficits in monkey. J. Neurophysiol. 23, 471–484. 10.1152/jn.1960.23.5.471 13770644

[B88] Miller E. K. Li L. Desimone R. (1991). A neural mechanism for working and recognition memory in inferior temporal cortex. Science 254, 1377–1379. 10.1126/science.1962197 1962197

[B89] Mishkin M. (1957). Effects of small frontal lesions on delayed alternation in monkeys. J. Neurophysiol. 20, 615–622. 10.1152/jn.1957.20.6.615 13476217

[B90] Mishkin M. Manning F. J. (1978). Non-spatial memory after selective prefrontal lesions in monkeys. Brain Res. 143, 313–323. 10.1016/0006-8993(78)90571-1 415803

[B91] Mishkin M. Pribram K. H. (1955). Analysis of the effects of frontal lesions in monkeys: I. Variations of delayed alternations. J. Comp. Physiol. Psychol. 48, 492–495. 10.1037/h0040318 13271624

[B92] Mishkin M. Pribram K. H. (1956). Analysis of the effects of frontal lesions in monkey. II. Variations of delayed response. J. Comp. Physiol. Psychol. 49, 36–40. 10.1037/h0040592 13295405

[B93] Moore T. Armstrong K. M. (2003). Selective gating of visual signals by microstimulation of frontal cortex. Nature 421, 370–373. 10.1038/nature01341 12540901

[B94] Murray J. D. Bernacchia A. Freedman D. J. Romo R. Wallis J. D. Cai X.. (2014). A hierarchy of intrinsic timescales across primate cortex. Nat. Neurosci. 17, 1661–1663. 10.1038/nn.3862 25383900 PMC4241138

[B95] Murray J. D. Jaramillo J. Wang X.-J. (2017). Working memory and decision-making in a frontoparietal circuit model. J. Neurosci. 37, 12167–12186. 10.1523/JNEUROSCI.0343-17.2017 29114071 PMC5729190

[B96] Newsome W. T. Pare,' E. B. (1988). A selective impairment of motion perception following lesions of the middle temporal visual area (MT). J. Neurosci. 8, 2201–2211. 10.1523/JNEUROSCI.08-06-02201.1988 3385495 PMC6569328

[B97] Orbach J. (1956). Immediate and chronic disturbances on the delayed response following transections of the frontal granular cortex in the monkey. J. Comp. Physiol. Psychol. 49, 46–51. 10.1037/h0048954 13295407

[B98] Orbach J. Fischer G. J. (1959). Bilateral resections of frontal granular cortex: factors influencing delayed response and discrimination performance in monkeys. JAMA Neurol. 1, 78–86. 10.1001/archneur.1959.03840010080010 14428978

[B99] Oscar-Berman M. Zurif E. B. Blumstein S. (1975). Effects of unilateral brain damage on the processing of speech sounds. Brain Lang. 2, 345–355. 10.1016/S0093-934X(75)80075-7 1182500

[B100] Ozdemir A. T. Lagler M. Lagoun S. Malagon-Vina H. Laszto'czi B. Klausberger T. (2020). Unexpected rule-changes in a working memory task shape the firing of histologically identified delay-tuned neurons in the prefrontal cortex. Cell Rep. 30, 1613–1626.e4. 10.1016/j.celrep.2019.12.102 32023473

[B101] Park J. C. Bae J. W. Kim J. Jung M. W. (2019). Dynamically changing neuronal activity supporting working memory for predictable and unpredictable durations. Sci. Rep. 9:15512. 10.1038/s41598-019-52017-8 31664169 PMC6820562

[B102] Passingham R. (1975). Delayed matching after selective prefrontal lesions in monkeys (*Macaca mulatta*). Brain Res. 92, 89–102. 10.1016/0006-8993(75)90529-6 809096

[B103] Passingham R. E. Wise S. P. (2012). The Neurobiology of the Prefrontal Cortex. Oxford: Oxford University Press.

[B104] Pasternak T. Greenlee M. W. (2005). Working memory in primate sensory systems. Nat. Rev. Neurosci 6, 97–107. 10.1038/nrn1603 15654324

[B105] Perrett D. I. Smith P. A. Potter D. D. Mistlin A. J. Head A. S. Milner A. D.. (1984). Neurones responsive to faces in the temporal cortex: studies of functional organization, sensitivity to identity and relation to perception. Hum. Neurobiol. 3, 197–208. 6526706

[B106] Petrides M. (1995). Impairments on nonspatial self-ordered and externally ordered working memory tasks after lesions of the mid-dorsal part of the lateral frontal cortex in the monkey. J. Neurosci. 15, 359–375. 10.1523/JNEUROSCI.15-01-00359.1995 7823141 PMC6578311

[B107] Petrides M. (2000). Dissociable roles of mid-dorsolateral prefrontal and anterior inferotemporal cortex in visual working memory. J. Neurosci. 20, 7496–7503. 10.1523/JNEUROSCI.20-19-07496.2000 11007909 PMC6772785

[B108] Petrides M. (2005). Lateral prefrontal cortex: architectonic and functional organization. Philos. Trans. R. Soc. Lond. Ser. B Biol. Sci. 360, 781–795. 10.1098/rstb.2005.1631 15937012 PMC1569489

[B109] Postle B. R. (2006). Working memory as an emergent property of the mind and brain. Neuroscience 139, 23–38. 10.1016/j.neuroscience.2005.06.005 16324795 PMC1428794

[B110] Pribram K. H. (1950). Some physical and pharmacological factors affecting delayed response performance of baboons following frontal lobotomy. J. Neurophysiol. 13, 373–382. 10.1152/jn.1950.13.5.373 14774751

[B111] Pribram K. H. Mishkin M. Rosvold H. E. Kaplan S. J. (1952). Effects on delayed-response performance of lesions of dorsolateral and ventromedial frontal cortex of baboons. J. Comp. Physiol. Psychol. 45, 565–575. 10.1037/h0061240 13000029

[B112] Reynolds J. H. Chelazzi L. Desimone R. (1999). Competitive mechanisms subserve attention in macaque areas V2 and V4. J. Neurosci. 19, 1736–1753. 10.1523/JNEUROSCI.19-05-01736.1999 10024360 PMC6782185

[B113] Riley M. R. Constantinidis C. (2015). Role of prefrontal persistent activity in working memory. Front. Syst. Neurosci. 9:181. 10.3389/fnsys.2015.00181 26778980 PMC4700146

[B114] Rolls E. T. (1984). Neurons in the cortex of the temporal lobe and in the amygdala of the monkey with responses selective for faces. Hum. Neurobiol. 3, 209–222. 10.1016/0166-4328(85)90062-2 6526707

[B115] Rosvold H. E. Delgado J. M. R. (1956). The effect on delayed-alternation test performance of stimulating or destroying electrically structures within the frontal lobes of the monkey's brain. J. Comp. Physiol. Psychol. 49, 365–372. 10.1037/h0087991 13345915

[B116] Roussy M. Luna R. Duong L. Corrigan B. Gulli R. A. Nogueira R.. (2021). Ketamine disrupts naturalistic coding of working memory in primate lateral prefrontal cortex networks. Mol. Psychiatry 1–16. 10.1038/s41380-021-01082-5 33981008 PMC8760073

[B117] Salazar R. F. Dotson N. M. Bressler S. L. Gray C. M. (2012). Content-specific fronto-parietal synchronization during visual working memory. Science 338, 1097–1100. 10.1126/science.1224000 23118014 PMC4038369

[B118] Salzman C. D. Newsome W. T. (1994). Neural mechanisms for forming a perceptual decision. Science 264, 231–237. 10.1126/science.8146653 8146653

[B119] Sawaguchi T. Goldman-Rakic P. S. (1991). D1 dopamine receptors in prefrontal cortex: involvement in working memory. Science 251, 947–950. 10.1126/science.1825731 1825731

[B120] Sawaguchi T. Iba M. (2001). Prefrontal cortical representation of visuospatial working memory in monkeys examined by local inactivation with muscimol. J. Neurophysiol. 86, 2041–2053. 10.1152/jn.2001.86.4.2041 11600660

[B121] Schaeffer D. J. Hori Y. Gilbert K. M. Joseph S. G. Menon R. S. Everling S. (2020). Divergence of rodent and primate medial frontal cortex functional connectivity. Proc. Natl. Acad. Sci. U.S.A. 117, 21681–21689. 10.1073/pnas.2003181117 32817555 PMC7474619

[B122] Schwedhelm P. Baldauf D. Treue S. (2020). The lateral prefrontal cortex of primates encodes stimulus colors and their behavioral relevance during a match-to-sample task. Sci. Rep. 10:4216. 10.1038/s41598-020-61171-3 32144331 PMC7060344

[B123] Scimeca J. M. Kiyonaga A. D'Esposito M. (2018). Reaffirming the sensory recruitment account of working memory. Trends Cogn. Sci. 22, 190–192. 10.1016/j.tics.2017.12.007 29475635

[B124] Selemon L. D. Goldman-Rakic P. S. (1988). Common cortical and subcortical targets of the dorsolateral prefrontal and posterior parietal cortices in the rhesus monkey: evidence for a distributed neural network subserving spatially guided behavior. J. Neurosci. 8, 4049–4068. 2846794 10.1523/JNEUROSCI.08-11-04049.1988 PMC6569486

[B125] Spaet T. Harlow H. F. (1943). Problem solution by monkeys following bilateral removal of the prefrontal areas. II. Delayed reaction problems involving use of the matching-from-sample method. J. Exp. Psychol. 32, 424–434. 10.1037/h0058008

[B126] Sreenivasan K. K. Curtis C. E. D'Esposito M. (2014). Revisiting the role of persistent neural activity during working memory. Trends Cogn. Sci. 18, 82–89. 10.1016/j.tics.2013.12.001 24439529 PMC3964018

[B127] Stamm J. S. Weber-Levine M. L. (1971). Delayed alternation impairments following selective prefrontal cortical ablations in monkeys. Exp. Neurol. 33, 263–278. 10.1016/0014-4886(71)90020-3 5001447

[B128] Suzuki M. Gottlieb J. (2013). Distinct neural mechanisms of distractor suppression in the frontal and parietal lobe. Nat. Neurosci. 16, 98–104. 10.1038/nn.3282 23242309 PMC4207121

[B129] Todorović D. (2020). What are visual illusions? Perception 49, 1128–1199. 10.1177/0301006620962279 33183136

[B130] Tong F. Pratte M. S. (2012). Decoding patterns of human brain activity. Annu. Rev. Psychol. 63, 483–509. 10.1146/annurev-psych-120710-100412 21943172 PMC7869795

[B131] Torres-Gomez S. Blonde J. D. Mendoza-Halliday D. Kuebler E. Everest M. Wang X.-J.. (2020). Changes in the proportion of inhibitory interneuron types from sensory to executive areas of the primate neocortex: implications for the origins of working memory representations. Cereb. Cortex 9:557. 10.1093/cercor/bhaa056 32227119 PMC8248585

[B132] Treue S. Martínez Trujillo J. C. (1999). Feature-based attention influences motion processing gain in macaque visual cortex. Nature 399, 575–579. 10.1038/21176 10376597

[B133] Tucker T. Kling A. (1967). Differential effects of early and late lesions of frontal granular cortex in the monkey. Brain Res. 5, 377–389. 10.1016/0006-8993(67)90045-5 4965537

[B134] Upright N. A. Brookshire S. W. Schnebelen W. Damatac C. G. Hof P. R. Browning P. G. F.. (2018). Behavioral effect of chemogenetic inhibition is directly related to receptor transduction levels in rhesus monkeys. J. Neurosci. 38, 7969–75. 10.1523/JNEUROSCI.1422-18.2018 30082415 PMC6136156

[B135] Uylings H. B. Groenewegen H. J. Kolb B. (2003). Do rats have a prefrontal cortex? Behav. Brain Res. 146, 3–17. 10.1016/j.bbr.2003.09.028 14643455

[B136] Wang X.-J. (1999). Synaptic basis of cortical persistent activity: the importance of NMDA receptors to working memory. J. Neurosci. 19, 9587–9603. 10.1523/JNEUROSCI.19-21-09587.1999 10531461 PMC6782911

[B137] Wang X.-J. (2009). A microcircuit model of prefrontal functions: ying and yang of reverberatory neurodynamics in cognition, in The Frontal Lobes, eds Risberg J. Grafman J. (Cambridge: Cambridge University Press), 92–127. 10.1017/CBO9780511545917.006

[B138] Wang X.-J. Tegne'r J. Constantinidis C. Goldman-Rakic P. S. (2004). Division of labor among distinct subtypes of inhibitory neurons in a cortical microcircuit of working memory. Proc. Natl. Acad. Sci. U.S.A. 101, 1368–1373. 10.1073/pnas.0305337101 14742867 PMC337059

[B139] Warren J. M. Divac I. (1972). Delayed response performance by rhesus monkeys with midprincipalis lesions. Psychon. Sci. 28, 146–148. 10.3758/BF03328689

[B140] Williams G. V. Goldman-Rakic P. S. (1995). Modulation of memory fields by dopamine D1 receptors in prefrontal cortex. Nature 376, 572–575. 10.1038/376572a0 7637804

[B141] Wimmer K. Nykamp D. Q. Constantinidis C. Compte A. (2014). Bump attractor dynamics in prefrontal cortex explains behavioral precision in spatial working memory. Nat. Neurosci. 17, 431–439. 10.1038/nn.3645 24487232

[B142] Xu Y. (2017). Reevaluating the sensory account of visual working memory storage. Trends Cogn. Sci. 21, 794–815. 10.1016/j.tics.2017.06.013 28774684

[B143] Yang S.-T. Wang M. Paspalas C. D. Crimins J. L. Altman M. T. Mazer J. A.. (2018). Core differences in synaptic signaling between primary visual and dorsolateral prefrontal cortex. Cereb. Cortex 28, 1458–1471. 10.1093/cercor/bhx357 29351585 PMC6041807

[B144] Yang W. Yuste R. (2017). *In vivo* imaging of neural activity. Nat. Methods 14, 349–359. 10.1038/nmeth.4230 28362436 PMC5903578

[B145] Yeterian E. Pandya D. (2010). Fiber pathways and cortical connections of preoccipital areas in rhesus monkeys. J. Comp. Neurol. 518, 3725–3375. 10.1002/cne.22420 20653031

[B146] Yeterian E. H. Pandya D. N. Tomaiuolo F. Petrides M. (2012). The cortical connectivity of the prefrontal cortex in the monkey brain. Cortex 48, 58–81. 10.1016/j.cortex.2011.03.004 21481342 PMC3161133

[B147] Zaitsev A. V. Povysheva N. V. Gonzalez-Burgos G. Lewis D. A. (2012). Electrophysiological classes of layer 2/3 pyramidal cells in monkey prefrontal cortex. J. Neurophysiol. 108, 595–609. 10.1152/jn.00859.2011 22496534 PMC3404790

[B148] Zaksas D. Pasternak T. (2006). Directional signals in the prefrontal cortex and in area MT during a working memory for visual motion task. J. Neurosci. 26, 11726–11742. 10.1523/JNEUROSCI.3420-06.2006 17093094 PMC6674769

